# Candida albicans-Induced Epithelial Damage Mediates Translocation through Intestinal Barriers

**DOI:** 10.1128/mBio.00915-18

**Published:** 2018-06-05

**Authors:** Stefanie Allert, Toni M. Förster, Carl-Magnus Svensson, Jonathan P. Richardson, Tony Pawlik, Betty Hebecker, Sven Rudolphi, Marc Juraschitz, Martin Schaller, Mariana Blagojevic, Joachim Morschhäuser, Marc Thilo Figge, Ilse D. Jacobsen, Julian R. Naglik, Lydia Kasper, Selene Mogavero, Bernhard Hube

**Affiliations:** a Department of Microbial Pathogenicity Mechanisms, Hans-Knöll-Institute, Jena, Germany; b Research Group Applied Systems Biology, Hans-Knöll-Institute, Jena, Germany; c Mucosal & Salivary Biology Division, Dental Institute, King’s College London, London, United Kingdom; d Research Group Microbial Immunology, Hans-Knöll-Institute, Jena, Germany; e Aberdeen Fungal Group, MRC Centre for Medical Mycology, University of Aberdeen, Aberdeen, United Kingdom; f Department of Dermatology, University Hospital Tübingen, Tübingen, Germany; g Institute for Molecular Infection Biology, University of Würzburg, Würzburg, Germany; h Faculty of Biological Sciences, Friedrich-Schiller-University Jena, Jena, Germany; i Institute of Microbiology, Friedrich-Schiller-University Jena, Jena, Germany; University of British Columbia

**Keywords:** *Candida albicans*, candidalysin, host cell damage, host cell invasion, intestinal barrier, necrosis, translocation

## Abstract

Life-threatening systemic infections often occur due to the translocation of pathogens across the gut barrier and into the bloodstream. While the microbial and host mechanisms permitting bacterial gut translocation are well characterized, these mechanisms are still unclear for fungal pathogens such as Candida albicans, a leading cause of nosocomial fungal bloodstream infections. In this study, we dissected the cellular mechanisms of translocation of C. albicans across intestinal epithelia *in vitro* and identified fungal genes associated with this process. We show that fungal translocation is a dynamic process initiated by invasion and followed by cellular damage and loss of epithelial integrity. A screen of >2,000 C. albicans deletion mutants identified genes required for cellular damage of and translocation across enterocytes. Correlation analysis suggests that hypha formation, barrier damage above a minimum threshold level, and a decreased epithelial integrity are required for efficient fungal translocation. Translocation occurs predominantly via a transcellular route, which is associated with fungus-induced necrotic epithelial damage, but not apoptotic cell death. The cytolytic peptide toxin of C. albicans, candidalysin, was found to be essential for damage of enterocytes and was a key factor in subsequent fungal translocation, suggesting that transcellular translocation of C. albicans through intestinal layers is mediated by candidalysin. However, fungal invasion and low-level translocation can also occur via non-transcellular routes in a candidalysin-independent manner. This is the first study showing translocation of a human-pathogenic fungus across the intestinal barrier being mediated by a peptide toxin.

## IMPORTANCE

Candida albicans, usually a harmless fungus colonizing human mucosae, can cause lethal bloodstream infections when it manages to translocate across the intestinal epithelium. This can result from antibiotic treatment, immune dysfunction, or intestinal damage (e.g., during surgery). However, fungal processes may also contribute. In this study, we investigated the translocation process of C. albicans using *in vitro* cell culture models. Translocation occurs as a stepwise process starting with invasion, followed by epithelial damage and loss of epithelial integrity. The ability to secrete candidalysin, a peptide toxin deriving from the hyphal protein Ece1, is key: C. albicans hyphae, secreting candidalysin, take advantage of a necrotic weakened epithelium to translocate through the intestinal layer.

## INTRODUCTION

Candida albicans is one of the predominant fungal species that colonizes the mucosal surfaces of most humans as a harmless member of the normal microbiota (1, 2). However, under certain circumstances, C. albicans can become pathogenic and cause diseases ranging from common superficial to severe systemic infections (3, 4). These life-threatening disseminated infections are initiated by endogenous colonizers, which translocate from mucosal surfaces into the bloodstream. Several studies have demonstrated that the intestinal population of C. albicans is the main source of disseminated candidiasis (5–11). However, in a healthy host, the intestinal epithelium, together with mucosal immune cells, constitute a stable barrier preventing C. albicans from translocating into the bloodstream. Host defense against translocation is further augmented by the physical barrier functions of the epithelial layer, with tight junctions and adherens junctions sealing paracellular spaces (12), and a mucous layer that protects the epithelium, thereby directly affecting C. albicans physiology and morphology (13). In addition, a balanced and diverse microbiota (14), the secretion of antimicrobial peptides (AMPs) (15), and the concerted activity of the innate and adaptive immune systems (14, 16) act to reduce hyphal burdens during periods of fungal overgrowth and restrict the fungus to the commensal (yeast) morphology. However, dysfunctions in these protective mechanisms can favor C. albicans translocation.

Predisposing conditions that trigger the commensal-to-pathogen shift and translocation of C. albicans are often iatrogenic. These conditions include an imbalance of the resident microbiota by use of antibiotics, a compromised immune system (e.g., due to chemotherapy or immunosuppressive therapy), or damage of epithelial barrier functions by iatrogenic impairment, for instance due to cytostatic treatment, surgery, or trauma (17, 18). Consequently, disseminated candidiasis is typically a nosocomial infection, and intensive care unit (ICU) patients are particularly susceptible to invasive C. albicans infections (19–21).

In principle, initiation of disseminated candidiasis requires at least one of four events: (i) entry of C. albicans cells by direct invasion of epithelial cells (ECs) from intestinal mucosal surfaces into blood capillaries or vessels; (ii) indirect translocation of C. albicans cells phagocytosed by host immune cells (“sampling”); (iii) direct damage of mucosal barriers, for example due to surgery, polytrauma, or drug treatment; or (iv) spread from fungal biofilms established on medical devices such as catheters (22, 23).

Translocation of C. albicans in murine models requires a combination of increased fungal colonization via removal of the bacterial microbiota, neutropenia, and intestinal barrier dysfunction in order to establish disseminated disease (24). Importantly, infrequent fungal translocation may also occur without epithelial damage under conditions of enhanced fungal colonization following antibiotic pretreatment (23), and it does not always result in systemic disease in immunocompetent mice (25).

Although dissemination of C. albicans from the intestinal tract has been studied *in vivo* (5, 24–27), the molecular mechanism of C. albicans translocation across intestinal barriers via epithelial invasion and the fungal attributes required for this process are poorly characterized. Pathogenic interactions of C. albicans with ECs can be divided into three stages: adhesion, invasion, and damage (28–31). Each of these steps requires hypha formation and the expression of hypha-associated genes. For example, hypha-associated expression of *ALS3* permits not only adhesion (32) but also induced endocytosis (33) and iron acquisition (34). Expression of *ECE1* (encoding the peptide toxin candidalysin) is essential for epithelial damage (35) and is the missing link between hypha formation and host cell damage (36).

In this study, we used an *in vitro* translocation model to characterize the events associated with C. albicans translocation through an intact intestinal barrier. We show that translocation in this model mainly occurs via a transcellular route associated with fungus-induced necrotic, but not apoptotic, cell death. A screen of >2,000 C. albicans gene deletion mutants identified several genes associated with intestinal epithelial damage. Selected damage-defective mutants were analyzed for their impact on epithelial barrier function and translocation. Our data suggest that C. albicans transcellular translocation through intestinal layers requires candidalysin-induced epithelial damage. This is the first study that shows a peptide toxin-mediated translocation of a human-pathogenic fungus.

## RESULTS

### Dynamics of fungal invasion, epithelial damage, and loss of epithelial barrier integrity during translocation of Candida albicans through intestinal epithelial layers.

**FIG 1:**
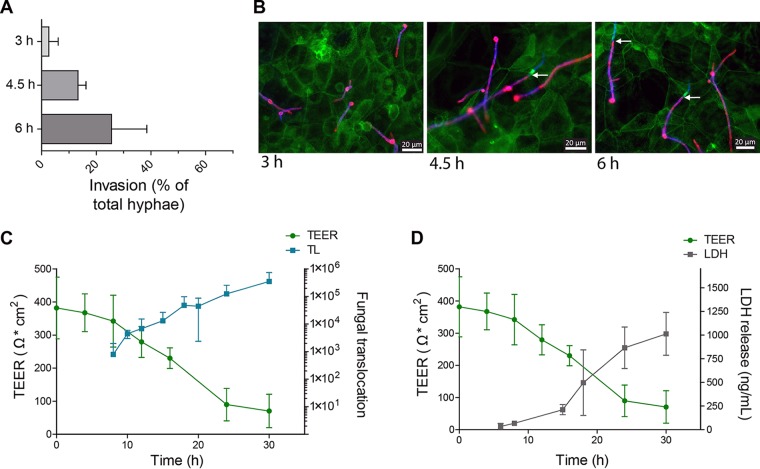
Infection of C2BBe1 IECs with wild-type C. albicans. (A) Invasion of C. albicans into C2BBe1 cells at 3 h, 4.5 h, and 6 h p.i. was quantified by differential fluorescence microscopy staining. The percentage of invasive hyphae relative to total C. albicans visible hyphae is shown. (B) Representative fluorescence microscopy images of differential staining to quantify C. albicans invasion. Extracellular C. albicans (pink), C. albicans (blue), and actin (green) are indicated. The white arrows indicate the entry point of the invading hypha. (C) Quantification of C2BBe1 barrier integrity as measured by TEER, and fungal translocation (number of translocated cells) after infection with C. albicans SC5314. (D) Release of LDH from C2BBe1 cells after infection with C. albicans SC5314. Data are presented as means ± standard deviations (SD) (error bars) from at least three independent experiments.

A combination of predisposing factors, including damage to epithelial barriers acquired during surgery or polytrauma, contribute to disseminated candidiasis. However, translocation of C. albicans across epithelial barriers can also occur without iatrogenic or accidental epithelial damage. We hypothesized that this particular type of translocation from the gut into the bloodstream requires invasion into intestinal epithelial cells (IECs) that is associated with fungus-induced cellular damage and loss of epithelial barrier integrity. To investigate the dynamics of this process, we established a translocation assay using the C2BBe1 cell line, a subclone of the human intestinal cell line Caco-2 (37), in a transwell system, by modifying previous protocols (29, 38, 39). We infected C2BBe1 enterocytes with wild-type (WT) C. albicans and monitored fungal invasion (via differential staining), host cell damage (via release of epithelial lactate dehydrogenase [LDH]), loss of epithelial monolayer integrity (via quantification of TEER [transepithelial electrical resistance]), and translocation (via fungal burdens) (Fig. 1).

Low-level invasion (3.6%) of C. albicans into C2BBe1 cells was observed 3 h postinfection (p.i.) and steadily increased over time (Fig. 1A and B). The observed invasion did not correlate with a loss of epithelial barrier integrity, epithelial damage, or translocation during the early stages (<3 h) of infection (Fig. 1C and D). However, extensive epithelial damage, loss of barrier integrity, and translocation were observed between 8 h and 30 h p.i. (Fig. 1C and D).

Previous studies have shown that invasion of C. albicans into differentiated enterocytes (Caco-2) is a hypha-dependent, fungus-driven process that occurs via active penetration, but not induced endocytosis, in contrast to other epithelial cells such as oral ECs (29, 40). To investigate whether the same was also true for the invasion of C. albicans into the C2BBe1 subclone, differentiated C2BBe1 cells were treated with the actin polymerization inhibitor cytochalasin D, which blocks induced endocytosis. Cytochalasin D did not impair invasion of C. albicans into C2BBe1 cells, and UV-killed hyphae, which are endocytosed by oral ECs (29), were not endocytosed by C2BBe1 cells (see Fig. S1 in the supplemental material). Therefore, invasion into differentiated C2BBe1 intestinal cells is a fungus-driven process.

10.1128/mBio.00915-18.1 FIG S1  Induced endocytosis does not contribute to the overall invasiveness of C. albicans into C2BBe1 cells. Invasion of C2BBe1 IECs by wild-type (Wt) C. albicans SC5314 (in the presence of 0.5 µM cytochalasin D [CytD]) and UV-killed Wt C. albicans hyphae. Data are presented as means ± SD from at least three independent experiments. Statistical significance: *, *P* ≤ 0.05. Download FIG S1, TIF file, 3.2 MB. Copyright © 2018 Allert et al. 2018 Allert et al. This content is distributed under the terms of the Creative Commons Attribution 4.0 International license.

### Large-scale screening of C. albicans mutant libraries identifies genes important for epithelial damage.

**FIG 2:**
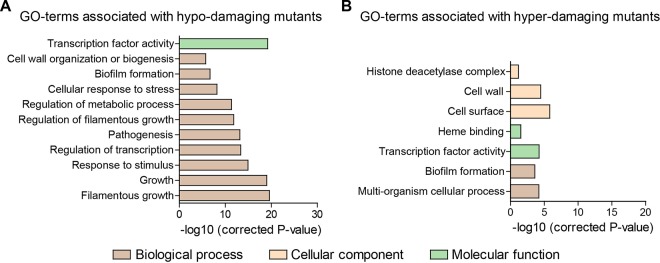
Gene Ontology (GO) term analysis of hypo- and hyperdamaging mutants. Gene deletions associated with significantly decreased (A) or increased (B) damage.

Since *in vitro* translocation of C. albicans correlated with cytotoxicity, we investigated whether C. albicans factors necessary for damage of IECs might also play a role in fungal translocation. Therefore, we screened three C. albicans gene deletion mutant libraries (41–43) for their ability to damage Caco-2 IECs by quantifying LDH levels. A total of 2,034 C. albicans gene deletion mutants were screened, including 1,165 homozygous open reading frame (ORF) deletions (approximately 20% of all annotated C. albicans genes [Data Set S1]). These included genes required for a broad spectrum of biological processes and potential virulence-associated traits (41), including transcriptional regulators (43). In total, we identified 172 C. albicans gene deletion mutants that caused significantly less damage (<μ − 2σ) compared to their respective WT control (shown in red in Data Set S1 in the supplemental material; see Data Set S2 also). *In silico* Gene Ontology (GO) term analysis revealed that these genes are putatively involved in filamentation, pathogenesis, and stress responses (among other functions) (Fig. 2A). The *Candida* Genome Database (44) identified 67 of these genes as having unknown function (shown in blue in Data Set S2). We also identified 102 C. albicans gene deletion mutants that caused more damage (>µ + 2σ) to Caco-2 IECs compared to the WT (shown in green in Data Set S1; see Data Set S2 also). *In silico* analysis of the corresponding genes of this subgroup showed putative associations with biofilm formation and cell surface composition (among other functions) (Fig. 2B). Fifty-two of these genes encode proteins with unknown function (shown in blue in Data Set S2).

10.1128/mBio.00915-18.8 DATA SET S1  C. albicans mutants screened for epithelial damage using Caco-2 cells via the LDH assay. C. albicans mutant collections from Noble et al. (41), Homann et al. (43), and Nobile and Mitchell (42) were screened for their ability to damage Caco-2 cells 24 h p.i. by LDH release. The mean LDH release as a percentage of the high control (full lysis) is shown for each mutant strain tested. Download DATA SET S1, XLSX file, 0.1 MB. Copyright © 2018 Allert et al. 2018 Allert et al. This content is distributed under the terms of the Creative Commons Attribution 4.0 International license.

10.1128/mBio.00915-18.9 DATA SET S2  C. albicans hypo- and hyperdamaging mutants. C. albicans gene deletion mutants were screened for their ability to damage Caco-2 cells 24 h p.i. by LDH release. A total of 172 hypodamaging gene deletion mutants and 102 hyperdamaging gene deletion mutants were identified and ordered according to damage caused (percent of the Wt). Putative gene function, fungal morphology on Caco-2 cells, and growth in YPD broth at 30°C are presented as a percentage of the Wt. X, defective compared to the Wt. Download DATA SET S2, XLSX file, 0.04 MB. Copyright © 2018 Allert et al. 2018 Allert et al. This content is distributed under the terms of the Creative Commons Attribution 4.0 International license.

The cytotoxicity analysis was performed in parallel with general growth assays in YPD broth (see Materials and Methods) and morphology analysis on IECs (Data Set S2). C. albicans gene deletion mutants with a general growth defect in complete medium and mutants with severe morphological defects were excluded from further analysis, since such phenotypes were expected to lead to unspecific or morphology-related reductions in damage. Indeed, the majority of gene deletion mutants that were compromised for hypha formation were also severely attenuated in their ability to cause damage (Data Set S2). Of the 172 gene deletion mutants identified that were hypodamaging to Caco-2 IECs, 38 mutants with no obvious growth or filamentation defects were selected for further analysis in epithelial damage assays using differentiated C2BBe1 cells (shown in orange in Data Set S2).

On the basis of these data, nine genes were selected for further analysis: *PRN4*, orf19.2797, *NPR2*, *AAF1*, *HMA1*, *TEA1*, orf19.3335, *PEP12*, and *ECE1* (Table S1). Selection criteria included (i) previously uncharacterized genes (*PRN4*, *NPR2*, orf19.2797, *HMA1*, *TEA1*, and orf19.3335), (ii) genes uncharacterized for interaction with IECs (*AAF1*, *PEP12*, and *ECE1*) (35, 45–47), and/or (iii) genes with putative functions, including urea transport, transcriptional regulation, ligand binding, and cytolytic toxicity (Table S2).

10.1128/mBio.00915-18.6 TABLE S1  C. albicans mutants analyzed in this study. Download TABLE S1, DOCX file, 0.05 MB. Copyright © 2018 Allert et al. 2018 Allert et al. This content is distributed under the terms of the Creative Commons Attribution 4.0 International license.

10.1128/mBio.00915-18.7 TABLE S2  C. albicans mutants analyzed for cellular damage, impact on epithelial integrity, and fungal translocation. Download TABLE S2, DOCX file, 0.1 MB. Copyright © 2018 Allert et al. 2018 Allert et al. This content is distributed under the terms of the Creative Commons Attribution 4.0 International license.

**FIG 3:**
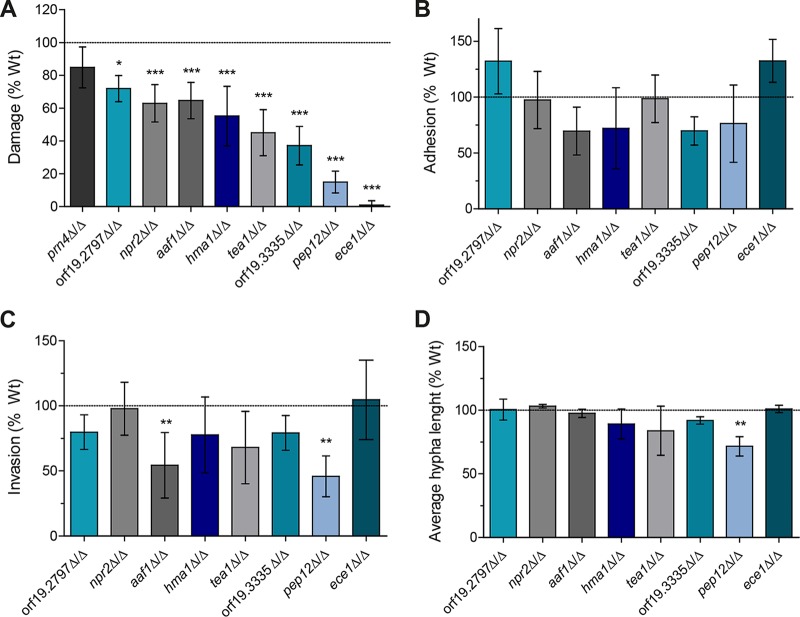
*In vitro* characterization of selected C. albicans mutants on C2BBe1 IECs. (A) C2BBe1 IECs were infected with selected C. albicans mutants, and cellular damage 24 h p.i. was quantified by LDH assay. The mean cellular damage induced by wild-type C. albicans was 865 ± 219 ng/ml LDH (dotted line). (B) The adhesion of selected C. albicans mutant strains to C2BBe1 IECs 1 h p.i. was quantified as described in Materials and Methods. The mean adhesion of WT C. albicans to C2BBe1 cells was 9.2% ± 5.7% (dotted line). (C) The invasion of selected C. albicans mutant strains into C2BBe1 IECs 5 h p.i. was quantified as described in Materials and Methods. The mean invasion level of WT C. albicans was 12.3% ± 5.9% (dotted line). (D) C2BBe1 IECs were infected with selected C. albicans mutants, and their mean hyphal length 5 h p.i. was determined. The mean length of WT C. albicans hyphae was 77.8 ± 12.6 µm (dotted line). All values are presented as mean ± SD relative to the WT. Values that are statistically significantly different are indicated by asterisks as follows: *, *P* ≤ 0.05; **, *P* ≤ 0.01; ***, *P* ≤ 0.001.

Since it is frequently observed that the altered phenotypes of mutants identified in the screen were not due to targeted gene deletion, but rather unspecific genomic alterations, and to exclude differences between genetic backgrounds, all nine selected mutants were recreated in a *C*. *albicans* BWP17 genetic background (48). These mutants were characterized in more detail (below), including quantification of their hyphal length, adhesion, invasion potential, and damage (Fig. 3 and S2). All mutants were significantly impaired in their ability to cause damage to differentiated C2BBe1 cells 24 h p.i., quantified by LDH measurements (Fig. 3A), with the exception of the *prn4*Δ/Δ mutant, which exhibited WT levels of damage and was thus not further investigated. The degree of damage reduction varied from moderate (less than 50%; *npr2*Δ/Δ, orf19.2797Δ/Δ, *aaf1*Δ/Δ, and *hma1*Δ/Δ) to severe (more than 50%: *tea1*Δ/Δ, orf19.3335Δ/Δ, *pep12*Δ/Δ, and *ece1*Δ/Δ). Interestingly, almost every mutant showed a unique pattern of phenotypic defects potentially responsible for the reduced damage observed (Fig. 3). For example, invasion of IECs by the *aaf1*Δ/Δ and *pep12*Δ/Δ mutants was significantly reduced, the *pep12*Δ/Δ mutant had significantly shorter hyphae compared to the WT hyphae, while the *hma1*Δ/Δ and orf19.3335Δ/Δ mutants showed moderately reduced adhesion, invasion, and hyphal length. In contrast, the *ece1*Δ/Δ mutant did not show any obvious phenotypic alteration except reduced damage (Fig. 3C and D; Fig. S2).

10.1128/mBio.00915-18.2 FIG S2  Average hypha length of selected C. albicans mutants. (A) Mean hypha length after 5 h on plastic in DMEM at 37°C and 5% CO_2_. Wt C. albicans (BWP17+Clp30) was 124.8 ± 39.2 µm (dotted line) (*n* ≥ 3). Statistical significance: *, *P* ≤ 0.05; ***, *P* ≤ 0.001. Download FIG S2, TIF file, 3.5 MB. Copyright © 2018 Allert et al. 2018 Allert et al. This content is distributed under the terms of the Creative Commons Attribution 4.0 International license.

### Translocation of C. albicans through enterocytes is associated with damage and loss of epithelial barrier integrity.

Our initial experiments demonstrate that translocation of C. albicans through the intestinal layer is associated with cellular damage and loss of epithelial integrity (Fig. 1). Accordingly, we tested whether decreased levels of epithelial damage were associated with a lower level of epithelial translocation. Further, we compared the ability of a “normal”-damaging mutant (*bas1*Δ/Δ) and a hyperdamaging mutant (*snt1*Δ/Δ; shown in green in Data Set S1; see Data Set S2 also) to translocate across an epithelial barrier. Several well-characterized control strains were included in this analysis, including mutants lacking genes involved in hyphal morphogenesis, hyphal maintenance, biofilm formation, adhesion, protein processing, or secreted protease activity (Table S2). Furthermore, translocation through a blank insert in the absence of an EC layer was quantified to exclude translocation impairment independent of host cells (see Materials and Methods; Fig. S3A).

10.1128/mBio.00915-18.3 FIG S3  Control experiments for *Candida* strains analyzed in the *in vitro* translocation model. (A) Translocation of C. albicans gene deletion mutants across a transwell membrane in the absence of C2BBe1 IECs. Data are presented as a mean ratio of mutant to Wt blank translocation (*n* ≥ 3). The same blank translocation ability of mutant and Wt would result in a ratio of 1 (dotted line). (B) C. albicans gene deletion mutants were assessed for their sensitivity to zymolyase. (C) Growth curve analysis of C. albicans gene deletion mutant strains in YPD broth at 30°C. The mutants in all graphs are separated according to cluster analysis. (B and C) Data are presented as mean ± SD from at least 3 independent experiments. Statistical significance: *, *P* ≤ 0.05. Download FIG S3, TIF file, 23.5 MB. Copyright © 2018 Allert et al. 2018 Allert et al. This content is distributed under the terms of the Creative Commons Attribution 4.0 International license.

**FIG 4:**
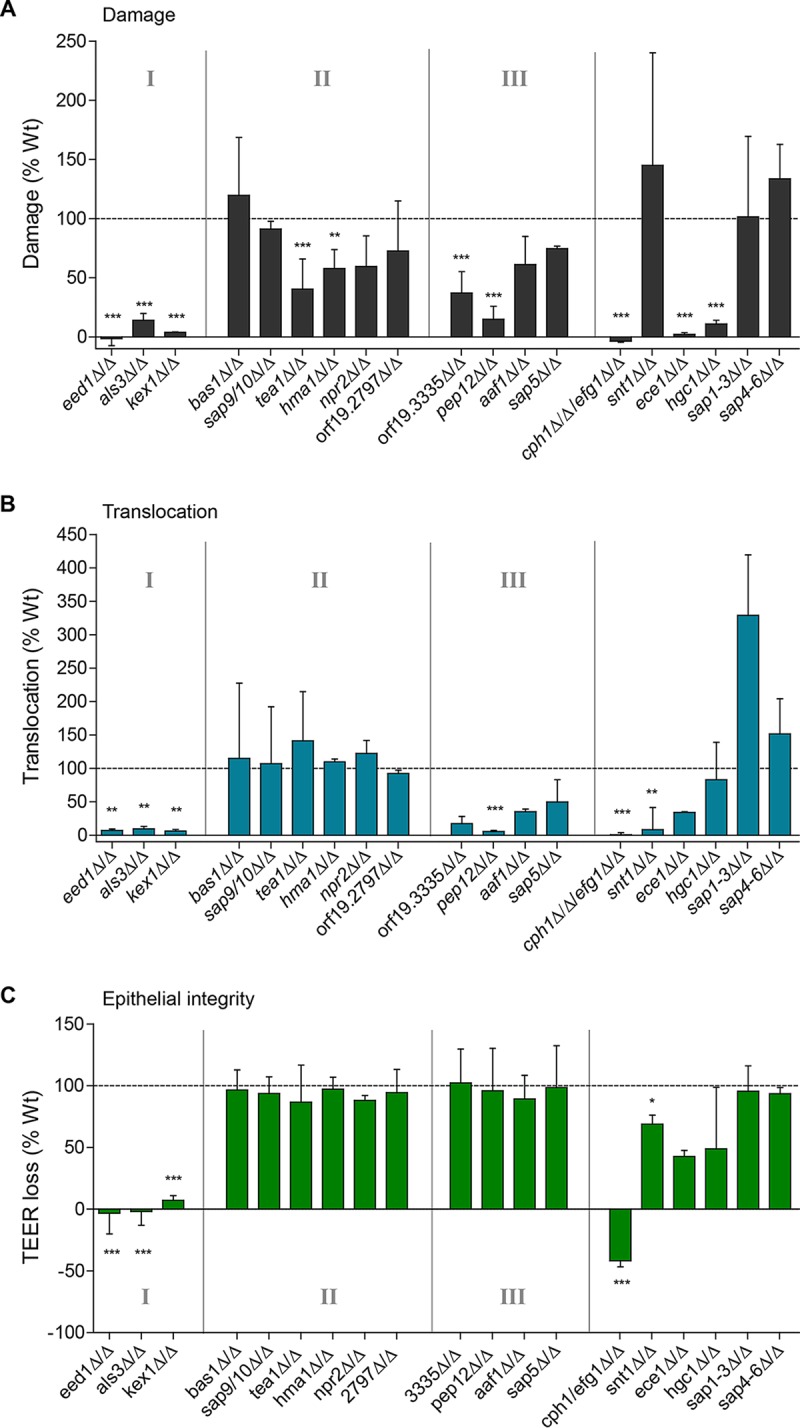
Characterization of damage, translocation, and loss of TEER by selected and control mutants. (A) C. albicans gene deletion mutants were analyzed for their ability to damage C2BBe1 IECs by LDH assay. (B) Translocation of C. albicans gene deletion mutants across a differentiated C2BBe1 intestinal epithelial barrier. (C) Assessment of C2BBe1 epithelial barrier integrity in response to C. albicans gene deletion mutants at 24 h p.i. as measured by loss of TEER. Data are expressed as TEER loss as a percentage of the wild-type C. albicans*-*infected cells. Strains are arranged in clusters (I to III) according to bioinformatic analysis. Cluster I exhibited low damage, translocation, and loss of TEER. Cluster II contains wild-type-like mutants. Cluster III exhibited low damage and translocation but wild-type-like loss of TEER. All values are presented as median plus range relative to the WT (dotted line). Statistical significance: *, *P* ≤ 0.05; **, *P* ≤ 0.01; ***, *P* ≤ 0.001.

Figure 4 summarizes the epithelial damage, fungal translocation, and epithelial integrity data for all 19 mutants. To identify correlations between mutant phenotypes and the ability to translocate through epithelial layers, and thus to identify properties associated with C. albicans translocation, we used a bioinformatic approach. First, we applied a density-based spatial clustering with noise (DBSCAN) algorithm (49) to categorize C. albicans mutants into groups with similar behavior based on the three parameters “epithelial damage,” “epithelial integrity,” and “fungal translocation” (Fig. 4 and S4). This led to the arrangement of strains in three clusters: (i) cluster I strains showing low damage, low translocation rates, and almost unaltered TEER values; (ii) cluster II strains being similar to the wild type; and (iii) cluster III strains displaying low damage and translocation, but wild-type-like loss of TEER. However, some C. albicans mutants could not be assigned to any cluster and were unique in their behavior in the translocation model. Interestingly, the *snt1*Δ/Δ mutant, a member of the hyperdamaging group, was significantly attenuated in causing loss of epithelial integrity and translocation through epithelial layers, whereas the hypha-deficient *hgc1*Δ/Δ mutant caused significantly reduced damage but could still translocate, while epithelial integrity was reduced by approximately 50%. An *ece1*Δ/Δ mutant displayed a moderate loss of epithelial integrity (50%) compared to the WT, but it caused almost no damage and exhibited a 75% reduction in translocation. Finally, the unassigned SAP mutants *sap1*-*3*Δ/Δ and *sap4*-*6*Δ/Δ showed a very strong similarity to cluster II, except for increased translocation potential, which was related to an altered ability to cross a blank transwell.

10.1128/mBio.00915-18.4 FIG S4  Cluster analysis of C. albicans mutants. Assignment of C. albicans mutants into different clusters according to epithelial damage, epithelial integrity, and fungal translocation by bioinformatic cluster analysis using DBSCAN. Clusters are shown in panel A, and individual mutants are listed in the table in panel B. Download FIG S4, TIF file, 8.9 MB. Copyright © 2018 Allert et al. 2018 Allert et al. This content is distributed under the terms of the Creative Commons Attribution 4.0 International license.

**FIG 5:**
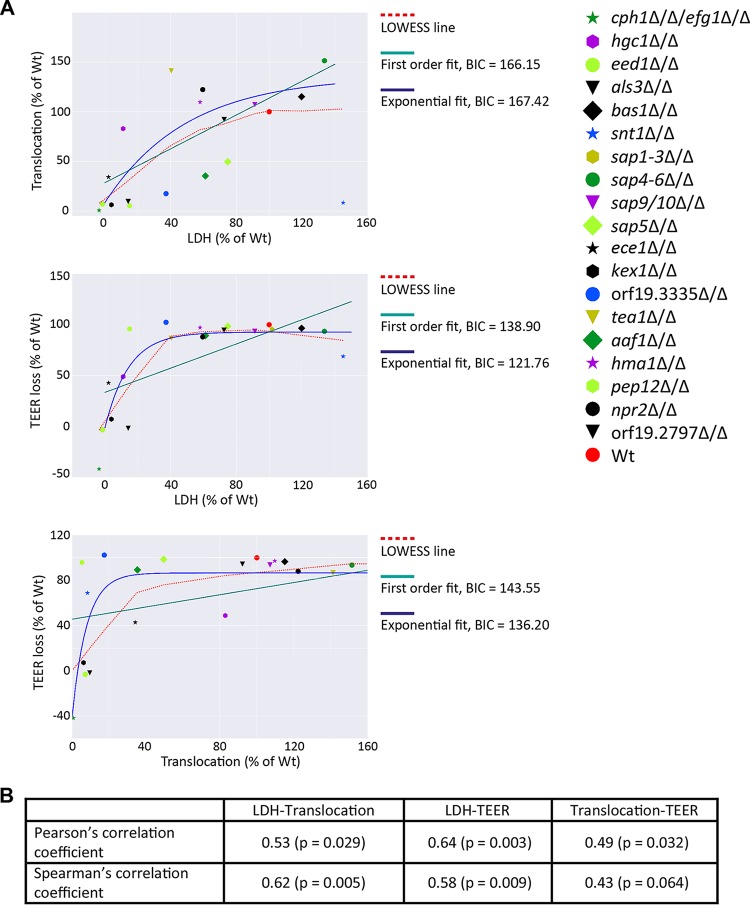
Bioinformatic analysis of C. albicans-induced epithelial damage, loss of epithelial integrity, and fungal translocation. Data obtained from WT and mutant strains of C. albicans for damage (LDH release), change in epithelial integrity (TEER), and translocation (CFU) were analyzed. (A) Pairwise correlation analysis of epithelial damage (LDH), barrier integrity (TEER), and translocation (CFU). In addition to the median value for each strain, the fit of a first-order polynomial and an exponential fit is shown together with a LOWESS line that describes a parameter-free smoothing to visualize the overall trend. The Bayesian information criterion (BIC) of each fit is given. Note that the *sap1-3*Δ/Δ strain is not visible, since it has extremely high translocation values; this data point was, however, taken into account in the calculation of correlations and in the curve fitting. (B) Correlation coefficients of the pairwise measurements presented. The respective *P* values are calculated using a two-tailed Student’s *t* test.

On the basis of these observations, a bioinformatic analysis was performed between pairings of the three parameters; “epithelial damage,” “epithelial integrity,” and “fungal translocation” to understand their reciprocal relationship. According to Pearson’s correlation analysis, all three parameters were significantly correlated, while according to Spearman’s correlation analysis, translocation and epithelial integrity (TEER) were not significantly correlated (Fig. 5). Both types of correlations were of moderate magnitude (~0.5, see Fig. 5B), which is explained by the strong variability among strains. For example, although translocation generally increases with increased epithelial damage (LDH), the *snt1*Δ/Δ mutant displays reduced translocation, even though it has the highest epithelial damage of all mutants. Next, to quantify any nonlinear behaviors in the correlations, we fitted a first-order polynomial and an exponential function of the form *y* = *Ae^−αx^* + *B* to each pair of measurements and evaluated the most appropriate model using the Bayesian information criterion (BIC) (50). The choices of independent measures (*x*) and dependent measures (*y*) are picked according to the respective *x*- and *y*-axes in Fig. 5A. We plotted the fitted polynomials and the LOWESS line (51) (i.e., a nonparametric smoothing that visualizes overall trends in noisy data) and provided the BIC values in Fig. 5A.

For epithelial damage and translocation, there was no improvement in the BIC using the exponential fit compared to a first-order polynomial, and the latter was observed to be close to the LOWESS line. This indicated a close-to-linear relationship between damage and translocation, suggesting that these two outcomes are most often the result of the same process or closely related processes. For epithelial damage and integrity, the LOWESS line and the exponential function tracked closely with another, which coincides with a considerable decrease in the BIC. This indicates that in the presence of epithelial damage, the epithelial integrity decreases rapidly to a minimum, independent of additional damage being induced. Last, translocation and epithelial integrity show the lowest correlation with a small improvement in BIC values using an exponential function. The increase is not as large as for epithelial damage and integrity, and the clear deviation from the LOWESS line indicates that neither function is able to track the structure of the data very well. The lack of significance in Spearman’s correlation coefficient can be explained by the structure of the data as seen in Fig. 5A. For strains with high translocation (>90% of WT), the epithelial integrity is consistently low, i.e., around WT levels (100% TEER loss), while for low translocation values (0 to 40% of WT), there is no clear structure in the epithelial integrity data. Pearson’s correlation coefficient takes into account that very high translocation indicates low epithelial integrity, while the nonsignificant Spearman’s correlation coefficient captures the lack of correlations within strains with low and high translocation. This reveals that while decreased epithelial integrity is a prerequisite for translocation, this does not predict fungal translocation (i.e., as seen with *pep12*Δ/Δ [Fig. 4]).

### Dissecting the potential routes of C. albicans translocation through enterocyte layers.

**FIG 6:**
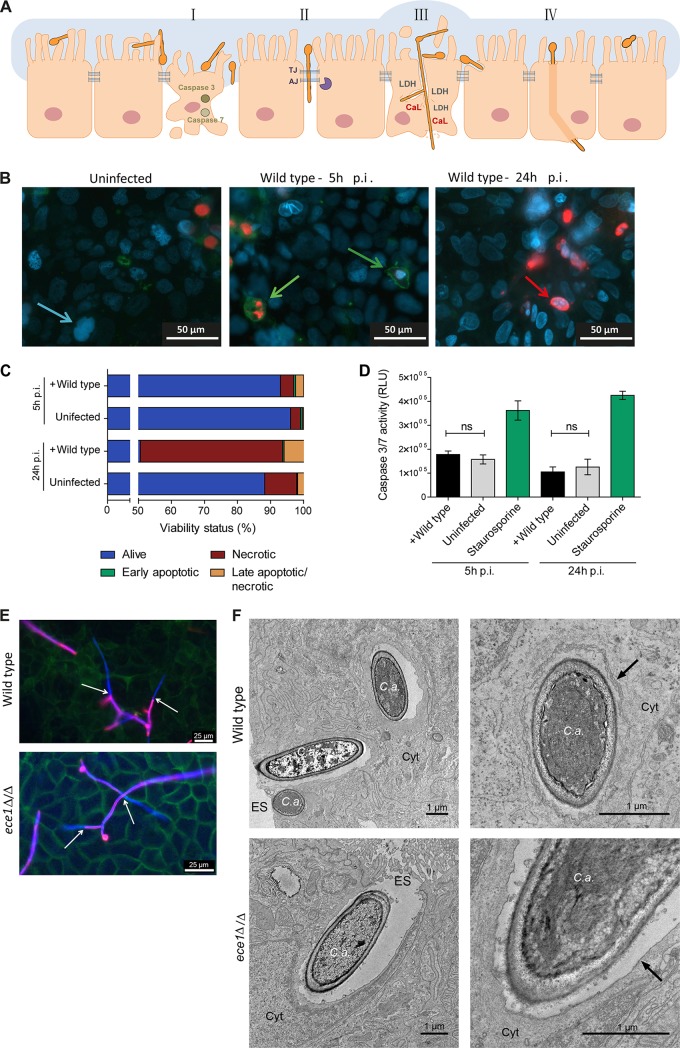
Possible translocation mechanisms of C. albicans through IECs. (A) Schematic representation of possible routes of C. albicans translocation. Possible routes of C. albicans translocation are shown as follows: I, apoptosis; II, paracellular; III, transcellular with damage; IV, transcellular without damage. TJ, tight junctions; AJ, adherens junctions; CaL, candidalysin. (B) C2BBe1 IECs were infected with WT C. albicans SC5314 for 5 h and 24 h and differentially stained. Living cells (Hoechst 33342) (blue), apoptotic cells (FITC-annexin V) (green), necrotic cells (ethidium homodimer III) (red), and late apoptotic/necrotic cells (red/green) are indicated by the color(s) indicated. Colored arrows point to examples of the stained cells. (C) A summary of statistical analysis is presented (mean ± SD; *n* = 3) that quantifies the proportion of live-apoptotic-necrotic staining observed in the images in panel B. (D) Quantification of caspase 3/7 activity in C2BBe1 IECs infected for 5 h and 24 h with C. albicans SC5314. Staurosporine was used as a positive control for the induction of apoptosis. Data are presented as means ± SD from three independent experiments. Caspase 3/7 activity is shown in relative light units (RLU). Values that are not significantly different (ns) are indicated. (E) Transcellular growth of WT C. albicans (BWP17+Clp30) and *ece1*Δ/Δ mutant hyphae through C2BBe1 IECs. C2BBe1 cells were infected with C. albicans and differentially stained at 6 h p.i. Extracellular C. albicans (pink), C. albicans (blue), and actin (green) are indicated. The white arrows show the point of invasion. (F) TEM images of C2BBe1 IECs infected with WT C. albicans (BWP17+Clp30) or *ece1*Δ/Δ mutant. The black arrows point to the host membrane. *C*.*a*., C. albicans; Cyt, cytoplasm; ES, extracellular space.

Our data and bioinformatic analyses suggest that while epithelial damage and loss of epithelial integrity are associated with translocation of C. albicans through epithelial barriers, this does not always predict the amount of fungal translocation. These analyses suggest two basic routes of gut translocation by C. albicans: paracellular (between adjacent IECs) and transcellular (through viable or nonviable enterocytes from the apical side to the basolateral side) (Fig. 6A, routes I to III). The latter would potentially be associated with EC death, which could either be necrotic or apoptotic.

Next, we investigated the role of epithelial apoptosis versus necrosis for transcellular translocation (Fig. 6). The contribution of enterocyte apoptosis following C. albicans infection was monitored by annexin V staining and fluorescence microscopy, as well as caspase 3/7 activity assays. We found that C. albicans does not induce apoptosis in C2BBe1 cells, even 24 h p.i. (Fig. 6B to D). In contrast, approximately 40% of IECs exhibited necrotic cell death 24 h p.i. in response to C. albicans, as indicated by ethidium homodimer III (EthD-III) staining (Fig. 6C). Therefore, necrotic cell death appears to be the major mechanism supporting C. albicans transcellular translocation in our model (Fig. 1, 3, and 6).

### Candidalysin is critical for intestinal epithelial damage and fungal translocation.

The secretion of the cytolytic peptide toxin candidalysin, encoded by the *ECE1* gene, is known to be critical for oral and vaginal EC damage (35, 52). Given that necrotic cell death appears to be the major mechanism supporting C. albicans transcellular translocation, we investigated the role of candidalysin in gut translocation in our *in vitro* model. We found that a C. albicans strain lacking *ECE1* was hypodamaging to IECs, was unable to translocate across the gut barrier, and was defective in reducing epithelial integrity (Fig. 3 and 4).

**FIG 7:**
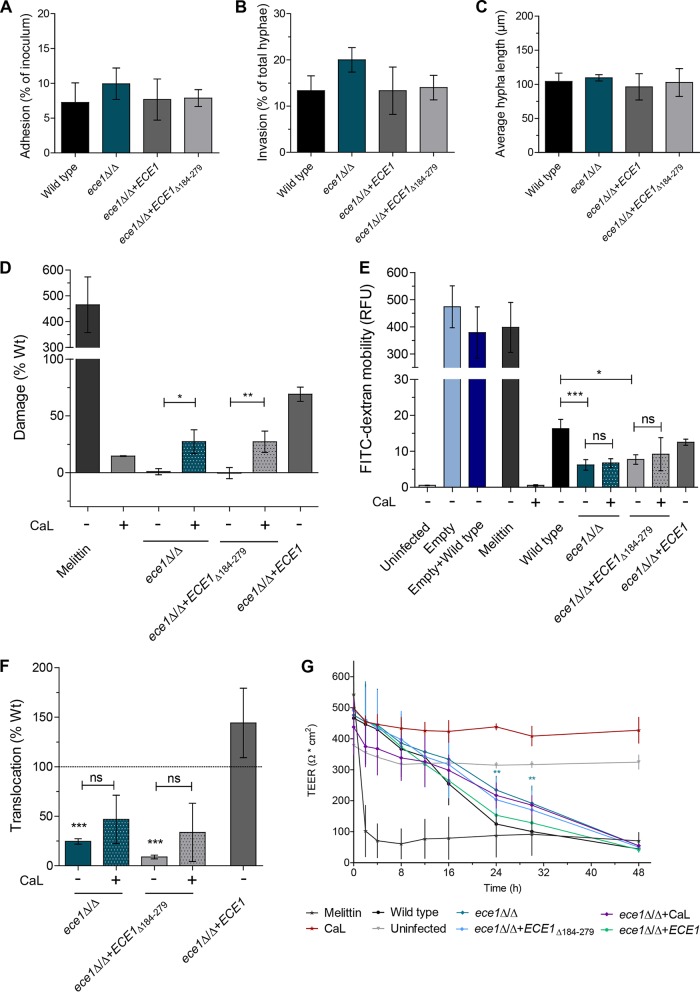
Interaction of C. albicans *ECE1* mutant strains with IECs. (A) Adhesion to C2BBe1 IECs as a percentage of inoculated cells of C. albicans *ECE1* mutant strains. (B) Invasion into C2BBe1 IECs as a percentage of total visible hyphae of *ECE1* mutant strains. (C) C2BBe1 cells were infected with *ECE1* mutant strains, and the mean hyphal length of infecting fungi 6 h p.i. was quantified. (D to G) Quantification of LDH (damage) (D), analysis of epithelial barrier integrity by dextran mobility assay (E), quantification of fungal translocation (F), and epithelial barrier integrity as measured by TEER in response to C. albicans *ECE1* mutant strains (G). In panels D to G, the application of exogenous candidalysin (CaL) toxin (70 µM) alone to C2BBe1 IECs and in combination with infecting *ECE1* mutant strains was assessed. Melittin (70 µM) was used as a positive control for damaging C2BBe1 cells. Data are presented as means ± SD relative to the WT from at least three independent experiments. Statistical significance: *, *P* ≤ 0.05; **, *P* ≤ 0.01; ***, *P* ≤ 0.001; ns, not significant.

To confirm that candidalysin was required for intestinal cell damage, we infected C2BBe1 cells with either a C. albicans mutant that lacked only the candidalysin-encoding region within *ECE1* (*ece1*Δ/Δ+*ECE1*_Δ184–279_ mutant) or with synthetic candidalysin toxin (35). Both the *ece1*Δ/Δ mutant and the *ece1*Δ/Δ+*ECE1*_Δ184–279_ mutant exhibited normal adhesion, epithelial invasion, and hyphal growth (Fig. 7A, B, and C) but were unable to induce epithelial damage (Fig. 7D), in contrast to the WT strain and a revertant strain expressing one WT copy of *ECE1* (*ece1*Δ/Δ+*ECE1* strain). Interestingly, the addition of synthetic candidalysin to IECs caused only minimal damage (Fig. 7). However, combined administration of candidalysin with the *ece1*Δ/Δ and *ece1*Δ/Δ+*ECE1*_Δ184–279_ mutants partially restored the damage capacity of these strains (Fig. 7D). This demonstrates that a combination of hypha formation and candidalysin secretion is required for optimal damage induction of IECs by C. albicans.

Next, we investigated the influence of candidalysin on intestinal barrier integrity by monitoring TEER and quantifying the diffusion of 4-kDa dextran polymers (Fig. 7E and G). The TEER values of C2BBe1 epithelium infected with WT C. albicans or an *ece1*Δ/Δ mutant constantly dropped over a time period of 48 h, but there was retention of TEER for the *ece1*Δ/Δ and *ece1*Δ/Δ+*ECE1*_Δ184–279_ mutants between 24 and 32 h p.i. (Fig. 7G). Accordingly, the dextran diffusion assay showed reduced barrier leakage in response to infection with the *ece1*Δ/Δ and *ece1*Δ/Δ+*ECE1*_Δ184–279_ mutants (Fig. 7E). In contrast to the damage assay, addition of synthetic candidalysin to C2BBe1 epithelium had no direct (single administration) or indirect (administration together with *ece1*Δ/Δ or *ece1*Δ/Δ+*ECE1*_Δ184–279_ mutant) effect on epithelial barrier function (Fig. 7E and G). As a means of comparison, we also administered melittin, a cytolytic peptide toxin in bee venom, as a positive control (53) in the damage and barrier integrity assays. Melittin caused high LDH release (Fig. 7D) and completely abolished epithelial barrier function (Fig. 7E and G).

As an expected consequence of reduced EC damage and reduced loss of integrity, translocation of *ece1*Δ/Δ and *ece1*Δ/Δ+*ECE1*_Δ184–279_ mutants was significantly reduced. We observed a trend toward higher translocation of *ece1*Δ/Δ and *ece1*Δ/Δ+*ECE1*_Δ184–279_ mutants when candidalysin was added during epithelial infection (Fig. 7F). Of note, while damage was almost abolished in the absence of candidalysin or *ECE1*, translocation of the respective mutants was still possible to a limited extent (approximately 25% of the WT level) (Fig. 4 and 7), emphasizing that translocation can occur without damage and independently of candidalysin. Therefore, we proposed a second route of transcellular translocation, which is not associated with EC damage (Fig. 6A, route IV). In this scenario, hyphae would invade ECs on the apical side and emerge on the basolateral side without causing host membrane damage and without causing release of cellular content (Fig. 6A, route IV). Fluorescence microscopy pictures clearly demonstrate that the hyphae of an *ece1*Δ/Δ mutant can invade and grow through IECs (Fig. 6E) without causing epithelial damage (Fig. 7D). Furthermore, transmission electron microscopy (TEM) pictures of invasive C. albicans hyphae show the presence of a host membrane surrounding hyphae in some pictures (Fig. 6F, black arrows), suggesting that transcellular translocation without membrane damage may be possible.

In conclusion, our data show that C. albicans is able to translocate through intact intestinal epithelial barriers predominantly via a damage-associated necrotic, but not apoptotic, transcellular route. Disturbance of epithelial integrity, cellular damage, and transcellular translocation requires a combination of fungal properties, including hypha formation and the secretion of candidalysin.

## DISCUSSION

The human gastrointestinal tract is colonized by a dense population of microorganisms, including bacteria and fungi. Although the gut epithelial layers and associated immune cells provide a functional barrier between these microbes and the host, microbial translocation is frequent, even in healthy individuals (54). However, in critically ill patients, translocation may lead to life-threatening infections. Several studies ascribe the major source of systemic candidiasis to the commensal C. albicans population of the human intestinal tract (8, 10, 55). However, we know little about how C. albicans translocates across intact intestinal epithelial barriers. In this study, we used an *in vitro* model and a reductionist approach to identify the C. albicans factors and host mechanisms involved in fungal translocation across the human gut barrier. While previous studies have focused on either C. albicans translocation (29, 38), C. albicans-dependent epithelial damage (29, 39), or epithelial integrity (39), our study analyzed all three processes over time to identify the fungal factors and host mechanisms associated with C. albicans translocation across the intact gut barrier. We demonstrate that C. albicans translocation is a dynamic fungus-driven process initiated by invasion (active penetration) and followed by cellular damage and loss of epithelial integrity. Experimental and bioinformatic correlation analyses indicated that epithelial damage and loss of epithelial integrity closely correlated with C. albicans translocation. Translocation occurs via a transcellular route, which is associated with fungus-induced necrotic epithelial damage, driven by the cytolytic peptide toxin candidalysin. However, fungal invasion and low-level translocation can also occur in a candidalysin-independent manner.

To identify fungal factors involved in C. albicans gut translocation, we screened >2,000 C. albicans gene deletion mutants. We identified 172 gene deletion mutants that were hypodamaging, including 38 mutants with no obvious growth or filamentation defects. Of these, eight mutants were selected for further analyses and subsequently found to have defects in adhesion, invasion, and hyphal length or potential defects in transcriptional and cellular regulation or protein trafficking (45–47). The main exception was a C. albicans *ECE1* gene deletion mutant, which had normal hypha formation/length and adhesion to and invasion of IECs but was defective in inducing epithelial damage. Interestingly, C. albicans *ECE1* encodes a cytolytic peptide toxin, candidalysin, which is critical for mucosal infections (35, 36).

Next, using a larger panel of C. albicans mutants, we determined that hypha formation was a key fungal attribute that promoted epithelial damage, loss of epithelial integrity, and translocation. However, translocation was not dependent upon hypha formation, as some mutants (e.g., *hgc1*Δ/Δ mutant [56]), which are generally locked in the yeast form or do not maintain hyphae, were still able to translocate across intestinal cells to a limited degree. Notably, though, the *hgc1*Δ/Δ mutant still expressed hypha-associated genes, including *ECE1* (data not shown), again potentially linking candidalysin to gut translocation. These findings may also explain contrasting data from *in vivo* translocation experiments, in which C. albicans gut translocation was shown to be both hypha dependent (24) and independent (25). Other proteins that contributed to C. albicans damage and translocation included Kex1, a Golgi protease involved in the processing of candidalysin (35, 57), and Als3, a glycosylphosphatidylinositol (GPI)-anchored adhesin and invasin that is the main trigger of induced endocytosis for certain types of epithelial and endothelial cells (33, 58). The *als3*Δ/Δ mutant had strong defects in damage, translocation, and disruption of epithelial integrity. However, since invasion into IECs occurs independently of induced endocytosis (29), we concluded that the observed phenotypes were mostly due to the severely reduced adhesion of this mutant (58) rather than direct action of Als3 on the gut barrier.

Our experimental findings were supported by bioinformatic approaches that also indicated a correlation between damage of IECs and translocation. Interestingly, a cluster of C. albicans mutants was identified with reduced epithelial damage but normal fungal translocation (Fig. 4, cluster II), indicating that only a certain level of damage induction is required to achieve efficient translocation. Furthermore, fungal translocation was not observed without reduction in TEER, strongly suggesting that disturbance of epithelial integrity is a prerequisite for translocation. However, a decrease of epithelial integrity does not necessarily lead to significant fungal translocation (as observed for *pep12*Δ/Δ and orf19.3335Δ/Δ mutants). Hence, TEER loss is necessary but not sufficient to cause fungal translocation. Thus, in agreement with Böhringer et al. (39), we propose that damage and destruction of epithelial integrity can be independent processes and that loss of epithelial integrity may be caused by necrotic cell damage (59) or opening of cell-cell connections (e.g., tight junctions). Epithelial damage and reduction of barrier integrity are likely coupled during necrosis, but loss of epithelial integrity can occur independently of necrosis.

One of the most intriguing findings of the study was the discovery that the hypha-associated peptide toxin candidalysin appears to be a crucial factor in mediating intestinal epithelial damage and fungal translocation. Notably, the combination of exogenous addition of candidalysin to an *ECE1*-deficient strain only partially restored WT damage and translocation levels, and the exogenous addition of candidalysin alone had little effect (Fig. 7). Several studies, including Moyes et al. (35), proposed a membrane-bound “invasion pocket” during hyphal invasion of epithelial cells (29, 60, 61) which was further verified for the C2BBe1 cells in this study (Fig. 6F). This invagination of the epithelial membrane at the site of hyphal invasion results in close contact of the fungus to host membranes and should allow an accumulation of candidalysin that may be required for full damage potential and subsequent translocation. Therefore, the exogenous addition of candidalysin probably does not fully mimic the natural secretion by hyphae within this invasion pocket and thus does not fully restore WT damage levels. It is less likely that the other non-candidalysin Ece1 peptides play a role in this setting, since when adding such peptides together with candidalysin, no increased damage was observed (data not shown); along the same line, adding candidalysin to a candidalysin-deficient strain (*ece1*Δ/Δ+*ECE1*_Δ184–279_) also only partially restored WT damage and translocation levels.

However, we noted that while the *ece1*Δ/Δ mutant was unable to damage ECs, it was still able to lower epithelial integrity and translocate across the intestinal barrier to some extent. This suggests that a damage-independent fungal factor(s) can also modulate epithelial integrity. Such a factor(s) probably contributes to the paracellular route of translocation by degrading cell-cell connections such as tight junctions or adherens junctions. Possible candidates contributing to the paracellular route of translocation are the secreted aspartic proteases (SAPs), which may promote degradation of the adherens junction protein E-cadherin (29, 62, 63). However, we found that mutants lacking different *SAP* genes (*sap1*-*3*Δ/Δ, *sap5*Δ/Δ, *sap4*-*6*Δ/Δ, and *sap9*/*10*Δ/Δ mutants) had no translocation defects. This is with the caveat that the *sap1*-*3*Δ/Δ mutant exhibited reduced translocation through blank transwell inserts (without C2BBe1 cells), and thus, we cannot exclude a minor role for SAPs in the paracellular route of translocation. In summary, our data demonstrate that C. albicans translocates across the intestinal epithelial barrier predominantly via the transcellular route, which requires hypha formation, active penetration (not induced endocytosis), candidalysin-induced epithelial damage, and cellular necrosis. However, C. albicans can also translocate via the paracellular route in a damage- and candidalysin-independent manner via currently unknown mechanisms.

While our *in vitro* data are likely to be reflective of the C. albicans translocation process *in vivo*, host-driven uptake and fungal translocation *in vivo* may also occur via specialized intestinal cells, in particular M cells associated with Peyer’s patches, which were not modeled into our assays. M cells are capable of endocytosing C. albicans (64) and are targeted by several pathogenic Gram-negative bacteria, including *Shigella*, *Salmonella*, and *Yersinia* spp. (65–67). Nevertheless, our study indicates that efficient C. albicans translocation can also occur in the absence of M cells, predominantly via candidalysin-mediated necrotic damage to the intestinal barrier. With this in mind, toxin-induced intestinal barrier dysfunction is also an important factor contributing to the pathogenicity of enteric bacteria. Clostridium perfringens produces a number of toxins that impair intestinal barrier function. These toxins include C. perfringens δ-toxin, a β-pore-forming-toxin, which is cytotoxic to Caco-2 cells and functions to reduce epithelial integrity (TEER) and increase permeability without altering tight junctions (68). In contrast, C. perfringens enterotoxin (CPE) directly attaches to and disintegrates tight junctions (claudin family), resulting in an increase in paracellular permeability across the epithelial barrier (69). Furthermore, the phospholipase C activity of C. perfringens alpha-toxin results in increased gut permeability, most likely due to the redistribution and/or degradation of tight junction proteins (70). Likewise, suilysin, a cholesterol-dependent cytolysin produced by the swine pathogen Streptococcus suis, is thought to promote bacterial translocation via epithelial damage induction, although the precise mechanisms are unclear (71). While many other bacteria are capable of translocating across the gut barrier, such as enteropathogenic Escherichia coli (EPEC), Campylobacter jejuni, and Salmonella enterica serotype Typhimurium, the mechanisms by which this occurs may not always be attributed to the function of toxins (72). Irrespective, the majority of enteric bacteria appear to translocate via the paracellular route rather than the transcellular route.

In summary, C. albicans translocation predominantly occurs via a transcellular route, associated with candidalysin-induced necrotic epithelial damage. However, invasion and low-level translocation can also occur in a candidalysin-independent manner, most likely via a paracellular route.

## MATERIALS AND METHODS

### Candida albicans strains and growth conditions.

All C. albicans strains used in this study are listed in Table S1 in the supplemental material. C. albicans strains were routinely grown on YPD broth/agar (1% yeast extract, 2% peptone, 2% d-glucose with or without 1.5% agar) at 30°C. For all experiments, C. albicans cells were cultured overnight in YPD broth at 30°C, shaking at 180 rpm. Cells from overnight cultures were collected by centrifugation and washed twice with phosphate-buffered saline (PBS), and the number of cells was adjusted as indicated.

### Generation of C. albicans mutant strains.

Gene deletions were performed as previously described (48). Deletion cassettes were generated by PCR by amplifying pFA-HIS1 and pFA-ARG4-based markers with the respective primers for the gene to be deleted (Data Set S3). C. albicans BWP17 was sequentially transformed with the generated deletion cassettes and then transformed with the CIp10 vector (73). All integrations were confirmed by PCR/sequencing. The *sap1-3*Δ/Δ and *sap4-6*Δ/Δ mutants were exceptions; these mutants were created by the Ura-blaster method (74–76).

10.1128/mBio.00915-18.10 DATA SET S3  Primers used in this study. Download DATA SET S3, XLSX file, 0.01 MB. Copyright © 2018 Allert et al. 2018 Allert et al. This content is distributed under the terms of the Creative Commons Attribution 4.0 International license.

### Culture and maintenance of IEC lines.

The intestinal epithelial Caco-2 subclone C2BBe1 (Caco-2 brush border expressing 1; ATCC CRL2102) (37) was routinely cultivated in Dulbecco modified Eagle medium (DMEM) (ThermoFisher Scientific) supplemented with 10% fetal calf serum (FCS) (Bio&Sell) and 10 µg/ml holotransferrin (Calbiochem Merck) in a humidified incubator at 37°C and 5% CO_2_. C2BBe1 cells were seeded in collagen I-coated wells (10 µg/ml collagen I for 2 h at room temperature [RT]; ThermoFisher Scientific). Transwell inserts (polycarbonate membrane with 5-µm pores; Corning) and 96-well plates were seeded with 2 × 10^4^ cells/well or insert, and glass coverslips were placed in 24-well plates with 1 × 10^5^ cells/well. C2BBe1 cells were cultured for 14 days for differentiation, with medium exchanged every 3 or 4 days.

The intestinal epithelial cell (IEC) line Caco-2 (ACC 169 from DSMZ) was cultivated in medium supplemented with 10% FCS and 1% NEAA (MEM [minimum essential medium] nonessential amino acids; Biochrom AG), for 2 days. All infections were performed in serum-free DMEM in a humidified incubator at 37°C and 5% CO_2_. This cell line was used only for the large-scale mutant screening (see “C. albicans large-scale mutant screening” below). For this purpose, Caco-2 cells were seeded in 96-well plates with 2 × 10^4^ cells/well and grown for 2 days to confluence.

### C. albicans large-scale mutant screening.

C. albicans strains were cultivated in YPD broth in 96-well plates and incubated for 24 h at 30°C and 180 rpm. On the next day, a 1:20 subculture was set up in fresh YPD and incubated overnight at 30°C and 180 rpm. The cultures were then diluted 1:10 in PBS. From this dilution, a 1:20 dilution in YPD was used to analyze growth at 30°C (see “Analysis of fungal growth” below), and a 1:20 dilution in serum-free DMEM was used to infect confluent Caco-2 cells. After 24 h of infection, damage was evaluated by quantifying the release of cytoplasmatic lactate dehydrogenase (LDH) [see “Quantification of cytotoxicity (LDH assay)” below]. In the large-scale mutant screening for damage, all values were compared to the values for a uninfected control treated with 0.25% Triton X-100 to obtain full lysis of the Caco-2 cells (full lysis control), and mutant values outside the 2σ range of the WT values were considered significantly different.

### Analysis of fungal growth.

Fungal growth was analyzed in 96-well plates in YPD broth. The C. albicans strains were added at a final density of 4 × 10^5^ cells/well. Growth was monitored by measuring the absorbance at 600 nm every 30 min for 2 days at 30°C in a microplate reader (Tecan).

### Quantification of adhesion, invasion, and hypha length.

C. albicans cells were added to 14-day-old C2BBe1 cells in 24-well plates to a final concentration of 1 × 10^5^ cells/well for adhesion assays and 5 × 10^4^ cells/well for invasion and hypha length assays. Control experiments for hypha length were performed the same way on plastic without C2BBe1 cells.

Adhesion of C. albicans to ECs was determined 1 h postinfection (p.i.). Nonattached C. albicans cells were removed by washing the cells three times with PBS. Samples were fixed with Histofix (Roth) for 15 min at RT or overnight at 4°C and subsequently rinsed three times with PBS. Adherent fungi were stained with calcofluor white (10 µg/ml in 0.1 M Tris-HCl [pH 9.0]; Sigma-Aldrich) for 20 min at RT in the dark. After the samples were washed three times with water, samples were mounted on glass slides with ProLong mountant (ThermoFisher Scientific) and analyzed by fluorescence microscopy. The number of adherent C. albicans cells was determined in about 100 random fields of a defined size (200 by 200 µm). Assuming an even distribution of *Candida* cells, the total number of adherent cells on the entire coverslip was calculated based on the number counted in the defined area. This number was expressed as a percentage of adhered cells versus inoculated C. albicans cells (see references 29 and 58 also). Invasion of C. albicans into differentiated C2BBe1 cells was analyzed by differential staining performed according to references 58 and 77 with the following minor modifications. Briefly, after 5 h of C. albicans infection, C2BBe1 cells were washed three times with PBS and fixed with Histofix. Extracellular, noninvasive fungal components were stained as follows. The cells were incubated with a primary antibody against C. albicans (1:2,000 in PBS) (rabbit anti-*Candida* BP1006; Acris Antibodies) for 1 h at 30°C, washed three times with PBS, and incubated with a secondary antibody (1:5,000 in PBS) (goat anti-rabbit antibody labeled with Alexa Fluor 488 [catalog no. A-11008; ThermoFisher Scientific]) for 1 h at 30°C. After the ECs were rinsed three times with PBS, they were permeabilized with 0.5% Triton X-100 for 10 min at RT and washed again three times with PBS. The actin cytoskeleton was stained with phalloidin-Alexa Fluor 594 (1:50 in PBS) (catalog no. A12381; ThermoFisher Scientific) for 1 h at 30°C. After the cells were washed again (three times with PBS), C. albicans cells were stained with calcofluor white as described above. After mounting, samples were visualized by fluorescence microscopy. The percentage of invasive C. albicans hyphae (only calcofluor white stained) was counted from at least 100 hyphae per strain in at least three independent experiments. The total hypha length was also recorded. For invasion experiments with cytochalasin D (Sigma-Aldrich), 1 µl of cytochalasin D or dimethyl sulfoxide (DMSO) (as solvent control) was added, to a final concentration of 0.5 µM 45 min prior to infection (78).

### Quantification of cytotoxicity (LDH assay).

Differentiated C2BBe1 cells in 96-well plates were infected for a defined time period with 8 × 10^4^ C. albicans cells/well or with candidalysin toxin (sequence, SIIGIIMGILGNIPQVIQIIMSIVKAFKGNK; ProteoGenix/Peptide Protein Research Ltd.) prepared in water and added to the desired final concentration. After coincubation, epithelial damage was quantified by measuring LDH release using a cytotoxicity detection kit (Roche) according to the manufacturer’s instructions. LDH isolated from rabbit muscle (Roche) was used to generate a standard curve. The background LDH value of uninfected C2BBe1 cells was subtracted, and the corrected LDH release was expressed as a percentage of the wild-type (WT) values unless otherwise stated. Cytotoxicity analysis was performed in triplicate and finally determined from at least three independent experiments.

### *In vitro* translocation model.

Differentiated C2BBe1 cells grown in transwell inserts (Fig. S5) were infected with 1 × 10^5^ C. albicans cells per transwell and/or candidalysin toxin for 24 h. Before and after incubation, transepithelial electrical resistance (TEER) values were measured using a volt-ohm meter (WPI). The resistance of a blank insert (≈120 Ω) was subtracted from each value. The absolute TEER loss (in ohms) of the respective WT was set at 100%, and the TEER loss of the mutant strains was expressed as a percentage of the WT value. After 24 h of infection, zymolyase (Amsbio) was added to the basolateral chamber to a final concentration of 20 U/ml and incubated for 2 h at 37°C and 5% CO_2_. Afterward, detached C. albicans hyphae were collected and plated onto YPD agar. Translocation was measured in triplicate in at least three independent experiments. Each C. albicans mutant was additionally tested for general growth, sensitivity against Zymolyase, and translocation independent of C2BBe1 (through a blank insert).

10.1128/mBio.00915-18.5 FIG S5  *In vitro* translocation model. (A) Schematic representation of the established *in vitro* translocation model. C2BBe1 cells were grown and differentiated on a porous membrane in a transwell system. After infection with C. albicans, changes in epithelial integrity were determined by measurement of TEER. Fungal translocation from the apical side of the transwell to the basolateral compartment was quantified after zymolyase treatment via CFU count. (B) Periodic acid-Schiff (PAS) staining of a cross section through a transwell membrane with differentiated C2BBe1 cells after Wt C. albicans infection. (C) TEM image of C2BBe1 cells after 14 days of differentiation in the transwell system. Cells form a confluent monolayer with brush border (arrow) on the apical surface. Download FIG S5, TIF file, 9.2 MB. Copyright © 2018 Allert et al. 2018 Allert et al. This content is distributed under the terms of the Creative Commons Attribution 4.0 International license.

To test for abnormal sensitivity toward zymolyase treatment, C. albicans strains were placed in a 96-well plate (1 × 10^5^ cells/well) in serum-free DMEM. After incubation for 3 h at 37°C and 5% CO_2_, zymolyase was added to a final concentration of 20 U/ml. Cells were incubated for 2 h, diluted in PBS, and plated onto YPD agar. Colonies were counted after 2 days and compared to the respective WT.

The translocation rate of C. albicans strains through blank, collagen I-coated transwell inserts was measured as indicated above. However, C. albicans strains were incubated in DMEM at 37°C and 5% CO_2_ for 3 h before addition to the inserts to induce filamentation and to reduce unspecific translocation of yeast cells. Blank translocation was used to correct the translocation rate through C2BBe1 cells. For each mutant, the efficiency of fungal translocation was calculated for each biological replicate. The percent translocation (TL) compared to the wild type (WT) was calculated as follows:

$$\%\text{TL }=(\frac{\text{TL of mutant}}{\text{TL of WT}})(\frac{\overset{¯}{\text{blank TL of WT}}}{\overset{¯}{\text{blank TL of mutant}}})\times 100$$

### Analysis of epithelial barrier integrity by dextran diffusion assay.

Dextran diffusion assays were performed by the method of Elamin et al. (79) with the following modifications. Briefly, fluorescein isothiocyanate (FITC)-labeled 4-kDa dextran beads (Sigma-Aldrich) were used to analyze the permeability of differentiated C2BBe1 monolayers infected with 1 × 10^5^ C. albicans cells/transwell insert and/or candidalysin for 24 h in serum- and pH indicator-free DMEM. After incubation, 30 µl of dextran beads (50 mg/ml in PBS) was added to the apical compartment and incubated for 3 h at 37°C and 5% CO_2_. Fluorescence (excitation wavelength of 490 nm and emission wavelength of 520 nm) of diffused dextran beads was measured in the basolateral transwell compartment. The background fluorescence of DMEM was subtracted from each value. Analyses were performed in duplicate and determined from at least three independent experiments.

### Analysis of apoptosis and necrosis.

Apoptosis and necrosis of differentiated C2BBe1 cells after infection with C. albicans were quantified using the apoptotic/necrotic/healthy cell detection kit (PromoKine) according to the manufacturer’s instructions. Differentiated C2BBe1 cells were infected with 1 × 10^5^ C. albicans cells/well for 5 h or 5 × 10^4^ cells/well for 24 h, then washed, and incubated for 15 min in the dark with FITC-annexin V, ethidium homodimer II, and Hoechst 33342. After the infected cells were washed, they were examined by fluorescence microscopy (Zeiss Axio Observer). Uninfected, differentiated C2BBe1 cells were used as a negative control. Treatment with 40% ethanol for 1 min was used as a positive control for necrosis, and treatment with 2 µM staurosporine was used as a positive control for apoptosis.

The activity of caspase 3 and 7 was quantified using a Caspase-Glo 3/7 assay (Promega) according to the manufacturer’s instructions. Differentiated C2BBe1 cells in 96-well plates were infected with 2 × 10^4^ C. albicans cells/well for 5 h or 24 h. The volume of medium in each well was adjusted to 50 µl, and 50 µl of Caspase-Glo 3/7 reagent was added to each well. After the addition of Caspase-Glo 3/7 reagent, luminescence measurements were taken every 10 min for 2 h at RT in the dark using a microplate reader. Each measurement was performed in duplicate, and the mean value was calculated from three independent experiments.

### Cluster analysis and correlation coefficient calculation.

Cluster analysis enabled C. albicans mutants to be assigned into groups with similar behavior regarding the three measurements “epithelial damage,” “epithelial integrity” and “fungal translocation,” as described by the values LDH, TEER and translocation. Clusters were identified in Python using the scikit-learn library (81) applying the density-based spatial clustering of applications with noise (DBSCAN) algorithm (49). A cluster was detected if (i) it contained at least three members and (ii) the points were closer than ε = 35 percentage points (pp) to at least one other member. DBSCAN automatically finds the number of clusters, and all points that are not considered part of any cluster were left unassigned.

For correlation coefficient calculation, two of the three parameters epithelial damage (LDH)/epithelial integrity (TEER)/fungal translocation were plotted against each other, using the median response of all mutants as a percentage of the WT. For epithelial integrity, a TEER loss of 100% reflects the drop in TEER caused by the WT, while the 100% LDH and translocation values are the epithelial damage and translocation of the WT. Both the Pearson and Spearman correlation coefficients between measurements were calculated. To visualize overall trends, the locally weighted scatterplot smoothing (LOWESS) line was used (51, 80). To evaluate the linearity, or lack thereof, we fitted a first-order polynomial and an exponential function of the formula *y* = *Ae*^−α*x*^+ *B*, representing linear and nonlinear relationships, respectively. We evaluated whether the exponential fit added any information about the pairwise relationship by calculating the Bayesian information criterion (BIC) for each fit. The BIC is defined as follows: BIC=(n)(logRSSn)+k(log n) where *n* is the number of fitted points, *k* is the number of parameters in the model, and RSS is the sum of squared residuals (50).

For the cluster analysis and correlation coefficient calculation, the median values of the data were used to reduce the effect of outliers.

### Transmission electron microscopy of C. albicans-infected C2BBe1 cells.

Differentiated C2BBe1 cells were infected with 2 × 10^5^ C. albicans cells/transwell. After coincubation for 24 h, cells were ﬁxed with Karnovsky ﬁxative (3% paraformaldehyde, 3.6% glutaraldehyde, pH 7.2) for 24 h at 4°C and postﬁxed with osmium solution (1% OsO_4_ [Roth] and 1.5% potassium ferrocyanide [Morphisto]) for 2 h at 4°C. Samples were rinsed with distilled water, block stained with uranyl acetate (2% in double-distilled water [ddH_2_O]), dehydrated in alcohol (stepwise 50 to 100%), and embedded in glycide ether (Serva) by polymerizing for 48 h at 60°C. Semithin sections of 1 µm were cut on an Ultracut Nova instrument (Leica) with a diamond knife and stained with toluidine blue stain (Morphisto) at 80°C. Regions of interest were ultrathin sectioned at 30 nm, mounted on copper grids, and analyzed using a Zeiss LIBRA 120 transmission electron microscope (Carl Zeiss, Inc.) operating at 120 kV.

### Statistical analysis.

All experiments were conducted, including technical duplicates or triplicates (from which the mean value was calculated) on at least three independent occasions (biological replicates). Diagrams show the mean of the biological replicates with standard deviation (SD). Statistics relative to the WT control were performed on log-transformed values by means of a one-way analysis of variance (ANOVA) test with a follow-up test for multiple comparisons (Dunnett’s correction). When comparisons were made between selected data sets, a Bonferroni’s correction was used instead. TEER time curves were statistically tested using two-way ANOVA.
