# Supplementary figures and images for: Candida albicans-Induced Epithelial Damage Mediates Translocation through Intestinal Barriers
Source: mBio. 2018 Jun 5;9(3):e00915-18. doi: 10.1128/mBio.00915-18 (PMC5989070; doi:10.1128/mBio.00915-18)

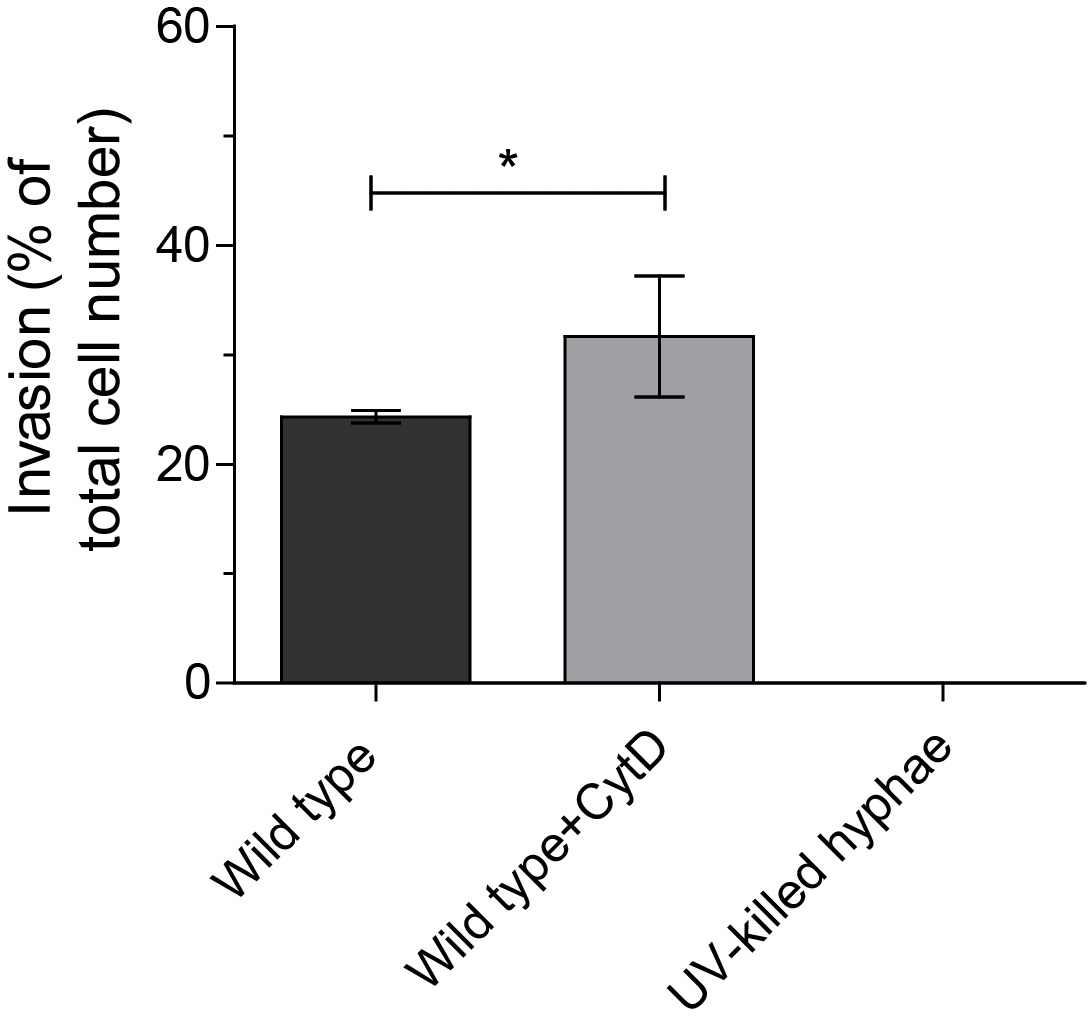

Supplement: FIG S1 [file mbo003183909sf1.tif]

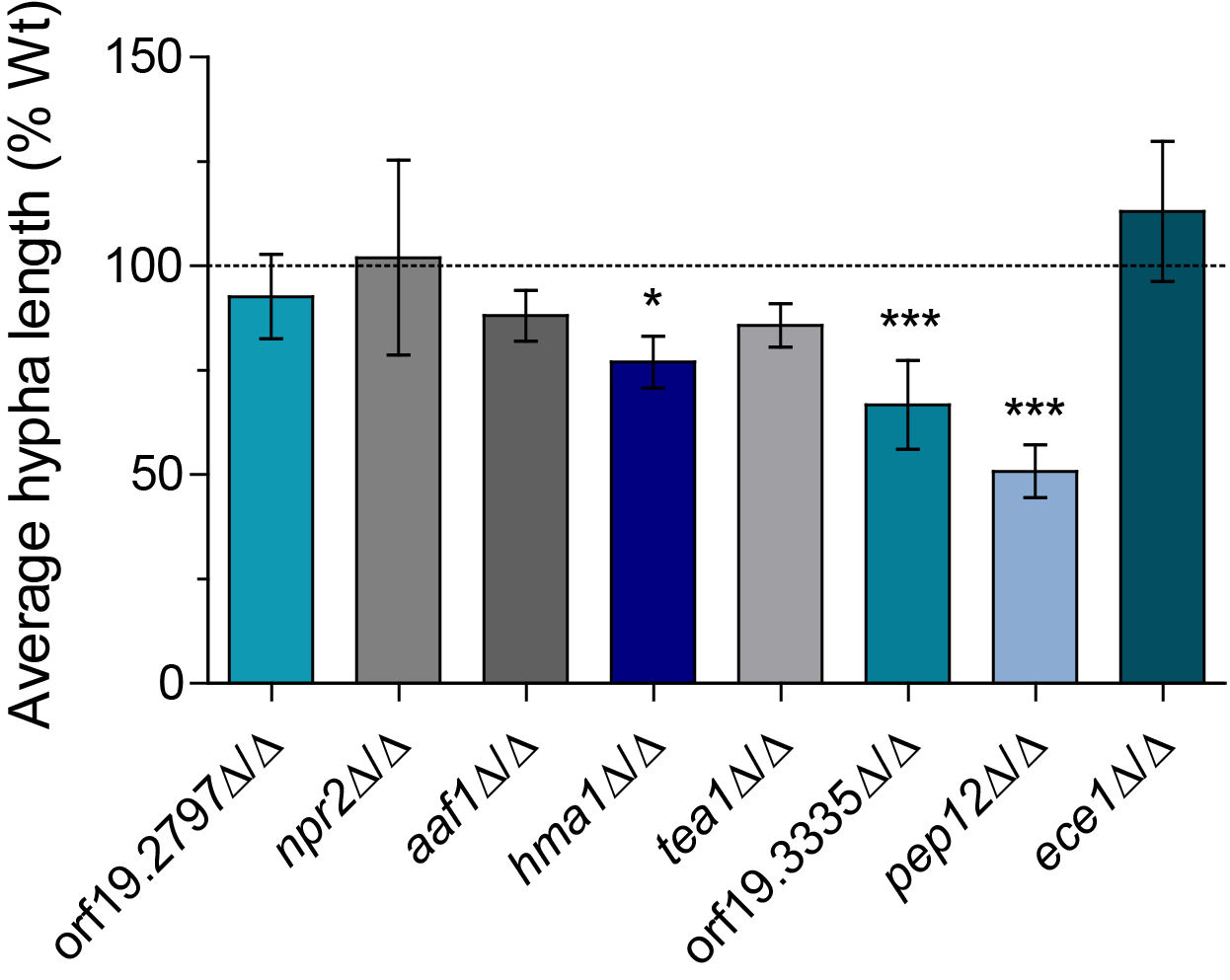

Supplement: FIG S2 [file mbo003183909sf2.tif]

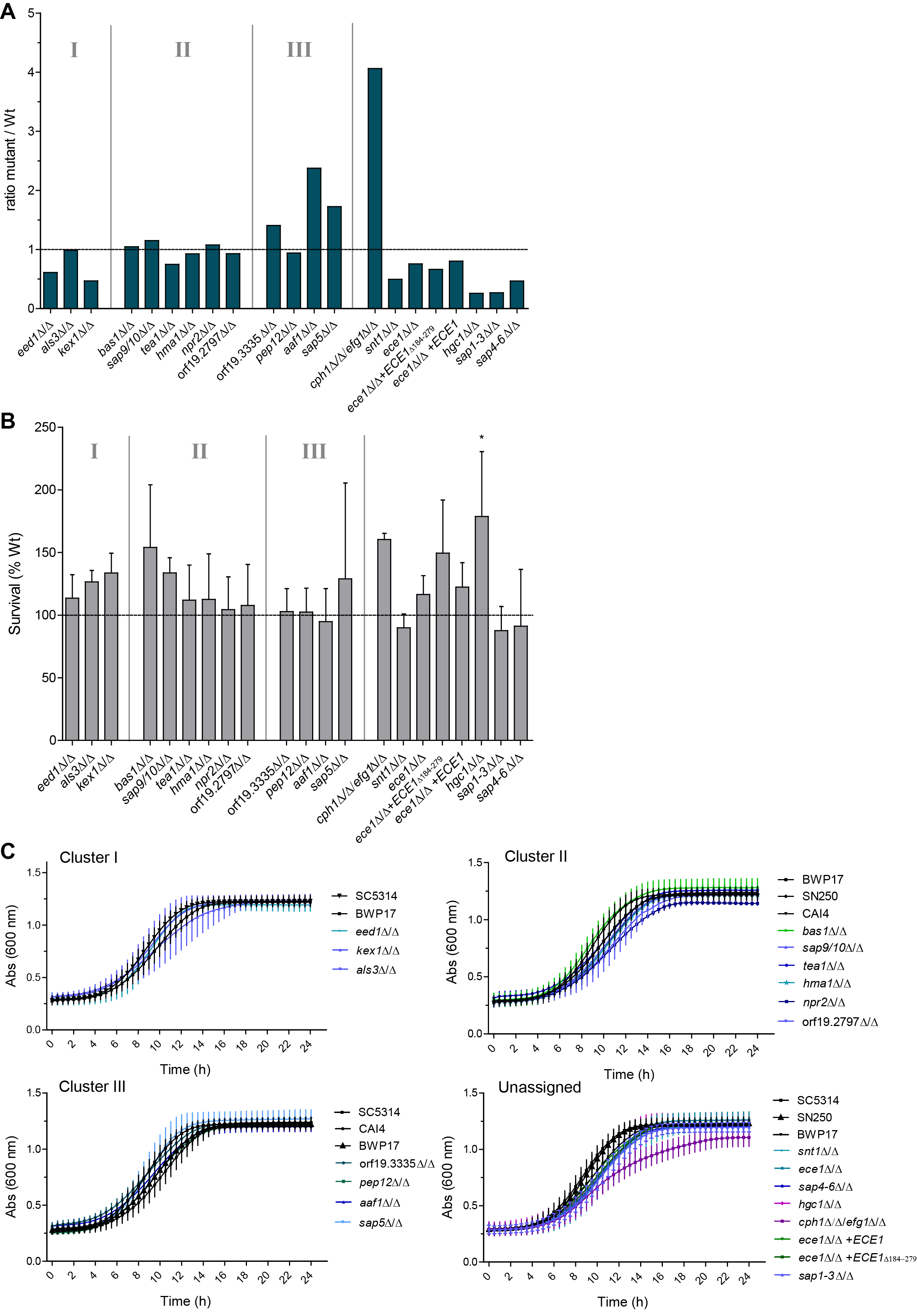

Supplement: FIG S3 [file mbo003183909sf3.tif]

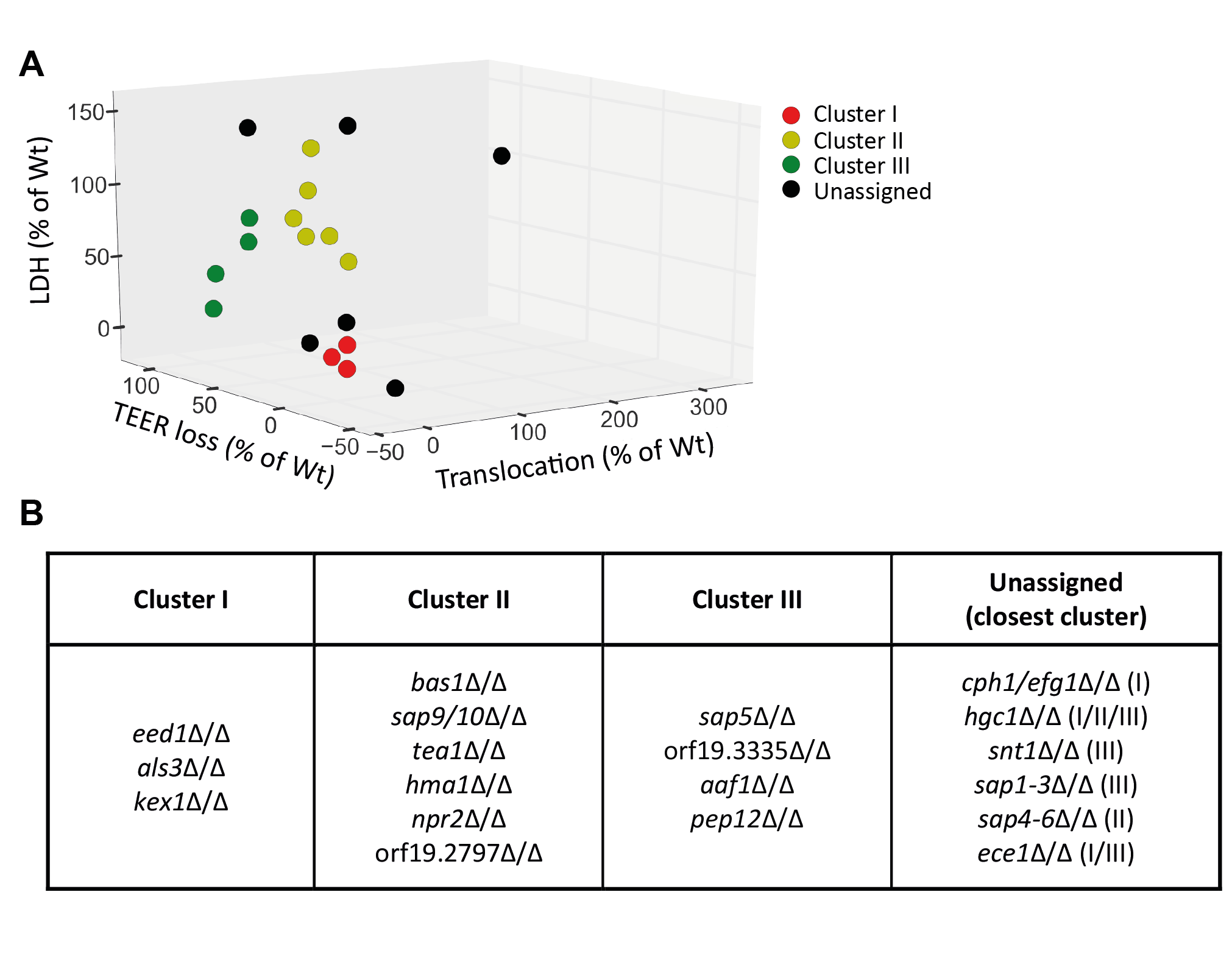

Supplement: FIG S4 [file mbo003183909sf4.tif]

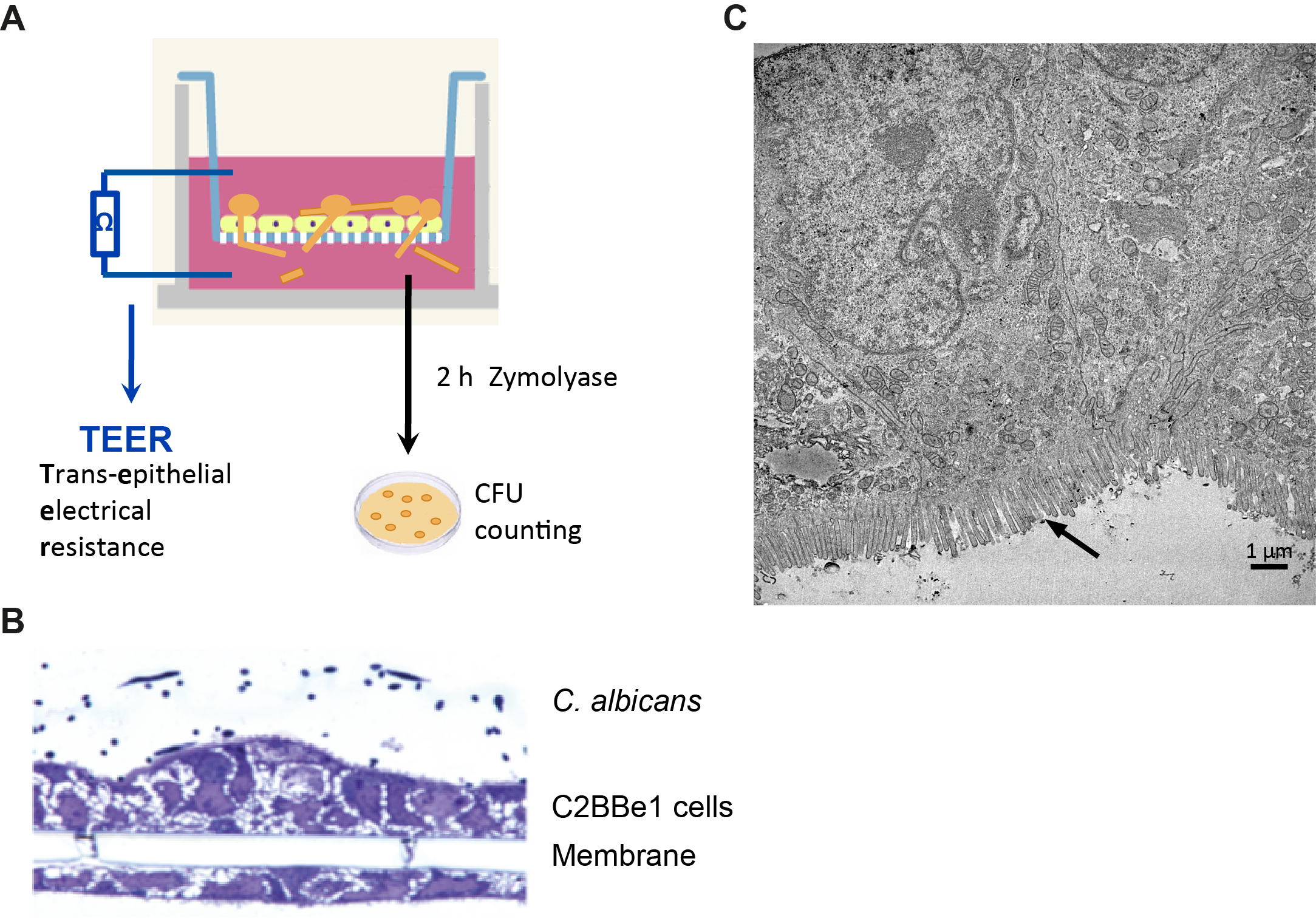

Supplement: FIG S5 [file mbo003183909sf5.tif]
