# Supplementary material for: Candida albicans-Induced Epithelial Damage Mediates Translocation through Intestinal Barriers
Source: mBio. 2018 Jun 5;9(3):e00915-18. doi: 10.1128/mBio.00915-18 (PMC5989070; doi:10.1128/mBio.00915-18)
Supplement: TABLE S1 [file mbo003183909st1.docx]

**Table S1**: *C. albicans* mutants analyzed in this study.

| ***C. albicans* strains** | **Parental strain** | **Relevant genotype** | **Source** |
| --- | --- | --- | --- |
| SC5314 |  | **wild type**, clinical isolate | Gillum *et al.* 1984 (1) |
| Mitchell Collection |  |  | Nobile & Mitchell *et al.* 2009 (2) |
| Noble Collection |  |  | Noble *et al.* 2010 (3) |
| Homann Collection |  |  | Homann *et al*. 2009 (4) |
| BWP17 |  | *ura3*::*imm434/ura3*::*imm434 iro1*::*imm434/iro1*::*imm434 his1*::*hisG/his1*::*hisG arg4*::*hisG/arg4*::*hisG* | Wilson *et al.* 1999 (5) |
| BWP17 + CIp30 | BWP17 | **wild type**, *RPS1*/*rps1*::(*HIS1 ARG4 URA3*) | Zakikhany *et al.* 2007 (6) |
| SN250 |  | **Noble wild type** (*leu2*::Cd*HIS1*/*leu2*::Cm*LEU2*) | Noble *et al.* 2010 (3) |
| CAI4 |  | *ura3*::*imm434*/*ura3*::*imm434* *iro1*::*imm434*/*iro1*::*imm434* | Fonzi *et al*. 1993 (7) |
| CAI4 + CIp10 | CAI4 | **wild type**, *RPS1*/*rps1*::*URA3* | Albrecht *et al.* 2006 (8) |
| orf19.3335∆/∆ | BWP17 | *orf19.3335*::*ARG4*/*orf19.3335*::*HIS1 RPS1*/*rps1*::*URA3* | this study |
| *tea1*∆/∆ | BWP17 | *orf19.6985*::*ARG4*/*orf19.6985*::*HIS1 RPS1*/*rps1*::*URA3* | this study |
| *aaf1*∆/∆ | BWP17 | *orf19.7436*::*ARG4*/*orf19.7436*::*HIS1 RPS1*/*rps1*::*URA3* | this study |
| *pep12*∆/∆ | BWP17 | *orf19.4292*::*ARG4*/*orf19.4292*::*HIS1 RPS1*/*rps1*::*URA3* | this study |
| *npr2*∆/∆ | BWP17 | *orf19.328*::*ARG4*/*orf19.328*::*HIS1 RPS1*/*rps1*::*URA3* | this study |
| orf19.2797∆/∆ | BWP17 | *orf19.2797*::*ARG4*/*orf19.2797*::*HIS1 RPS1*/*rps1*::*URA3* | this study |
| *hma1*∆/∆ | BWP17 | *orf19.2115*::*ARG4*/*orf19.2115*::*HIS1* *RPS1*/*rps1*::*URA3* | this study |
| *prn4*∆/∆ | BWP17 | *orf19.2461*::*ARG4*/*orf19.2461*::*HIS1 RPS1*/*rps1*::*URA3* | this study |
| *cph1*Δ/Δ */efg1*Δ/Δ | CAI4 | *cph1*::*hisG*/*cph1*::*hisG* *efg1*::*hisG*/*efg1*::*hisG*-*URA3*-*hisG* | Lo *et al.* 1997 (9) |
| *hgc1*Δ/Δ | BWP17 | *hgc1*::*ARG4*/*hgc1*::*HIS1 RPS1*/*rps1*::*URA3* | Zheng *et al.* 2004 (10) |
| *eed1*Δ/Δ | SC5314 | *eed1*::*FRT*/*eed1*::*FRT* | Martin *et al.* 2011 (11) |
| *brg1*Δ/Δ | SN250 | *brg1*::Cd*HIS1*/*brg1*::Cm*LEU1* | Noble *et al*. 2010 (3) |
| *ece1*Δ/Δ | BWP17 | *ece1*::*HIS1*/*ece1*::*ARG4* *RPS1*/*rps1*::*URA3* | Moyes *et al*. 2016 (12) |
| *ece1*Δ/Δ+*ECE1* | BWP17 | *ece1*::*HIS1*/*ece1*::*ARG4* *RPS1*/*rps1*::(*URA3 ECE1*) | Moyes *et al*. 2016 (12) |
| *ece1*∆/∆*+ECE1_∆184-279_* | BWP17 | *ece1*::*HIS1*/*ece1*::*ARG4* *RPS1*/*rps1*::(*URA3* *ECE1*Δ_184-279_ ) | Moyes *et al*. 2016 (12) |
| *sap1-3*Δ/Δ | CAI4 | *sap1*::*hisG*/*sap1*::*hisG* *sap2*::*hisG*/*sap2*::*hisG* *sap3*::*hisG*/*sap3*::*hisG* *RPS1*/*rps1*::*URA3* | Hube *et al*. 1997 (13); this study |
| *sap4-6*Δ/Δ | SC5314 | *sap6*::*hisG*/*sap6*::*hisG* *sap4*::*hisG*/*sap4*::*hisG* *sap5*::*hisG*/*sap5*::*hisG*  *ura3*::*imm434*/*ura3*::*imm434* *iro1*::*imm434*/*iro1*::*imm434* *RPS1*/*rps1*::*URA3* | Sanglard *et al*. 1997 (14);  this study |
| *sap9-10*Δ/Δ | CAI4 | *sap10*::*hisG*/*sap10*::*hisG* *sap9*::*hisG*/*sap9*::*hisG* *RPS1*/*rps1*::*URA3* | Schild *et al.* 2011 (15) |
| *sap5*Δ/Δ | SC5314 | *sap5*::*FRT*/*sap5*::*FRT* | Lermann *et al.* 2008 (16) |
| *als3*Δ/Δ | BWP17 | *ura3*::(*URA3 IRO1*)/*ura3*::*imm434* *als3*::*ARG4*/*als3*::*HIS1* | Nobile *et al*. 2006 (17) |
| *bas1*Δ/Δ | SN250 | *bas1*::Cd*HIS1*/*bas1*::Cm*LEU1* | Noble *et al*. 2010 (3) |
| *snt1*Δ/Δ | SN250 | *snt1*::Cd*HIS1*/*snt1*::Cm*LEU1* | Noble *et al*. 2010 (3) |
| *kex1*Δ/Δ | BWP17 | *kex1*::*HIS1*/*kex1*::*ARG4* *RPS1*/*rps1*::*URA3* | Moyes *et al.* 2016 (12) |

1. **Gillum AM, Tsay EY, Kirsch DR.** 1984. Isolation of the *Candida albicans* gene for orotidine-5'-phosphate decarboxylase by complementation of *S. cerevisiae* ura3 and *E. coli* pyrF mutations. Mol Gen Genet **198:**179-182.

2. **Nobile CJ, Mitchell AP.** 2009. Large-scale gene disruption using the UAU1 cassette. Methods Mol Biol **499:**175-194.

3. **Noble SM, French S, Kohn LA, Chen V, Johnson AD.** 2010. Systematic screens of a *Candida albicans* homozygous deletion library decouple morphogenetic switching and pathogenicity. Nat Genet **42:**590-598.

4. **Homann OR, Dea J, Noble SM, Johnson AD.** 2009. A phenotypic profile of the *Candida albicans* regulatory network. PLoS Genet **5:**e1000783.

5. **Wilson RB, Davis D, Mitchell AP.** 1999. Rapid hypothesis testing with *Candida albicans* through gene disruption with short homology regions. J Bacteriol **181:**1868-1874.

6. **Zakikhany K, Naglik JR, Schmidt-Westhausen A, Holland G, Schaller M, Hube B.** 2007. In vivo transcript profiling of *Candida albicans* identifies a gene essential for interepithelial dissemination. Cell Microbiol **9:**2938-2954.

7. **Fonzi WA, Irwin MY.** 1993. Isogenic strain construction and gene mapping in *Candida albicans*. Genetics **134:**717-728.

8. **Albrecht A, Felk A, Pichova I, Naglik JR, Schaller M, de Groot P, Maccallum D, Odds FC, Schafer W, Klis F, Monod M, Hube B.** 2006. Glycosylphosphatidylinositol-anchored proteases of *Candida albicans* target proteins necessary for both cellular processes and host-pathogen interactions. J Biol Chem **281:**688-694.

9. **Lo HJ, Kohler JR, DiDomenico B, Loebenberg D, Cacciapuoti A, Fink GR.** 1997. Nonfilamentous *C. albicans* mutants are avirulent. Cell **90:**939-949.

10. **Zheng X, Wang Y, Wang Y.** 2004. Hgc1, a novel hypha-specific G1 cyclin-related protein regulates *Candida albicans* hyphal morphogenesis. EMBO J **23:**1845-1856.

11. **Martin R, Moran GP, Jacobsen ID, Heyken A, Domey J, Sullivan DJ, Kurzai O, Hube B.** 2011. The *Candida albicans*-specific gene *EED1* encodes a key regulator of hyphal extension. PLoS One **6:**e18394.

12. **Moyes DL, Wilson D, Richardson JP, Mogavero S, Tang SX, Wernecke J, Hofs S, Gratacap RL, Robbins J, Runglall M, Murciano C, Blagojevic M, Thavaraj S, Forster TM, Hebecker B, Kasper L, Vizcay G, Iancu SI, Kichik N, Hader A, Kurzai O, Luo T, Kruger T, Kniemeyer O, Cota E, Bader O, Wheeler RT, Gutsmann T, Hube B, Naglik JR.** 2016. Candidalysin is a fungal peptide toxin critical for mucosal infection. Nature **532:**64-68.

13. **Hube B, Sanglard D, Odds FC, Hess D, Monod M, Schafer W, Brown AJ, Gow NA.** 1997. Disruption of each of the secreted aspartyl proteinase genes *SAP1*, *SAP2*, and *SAP3* of *Candida albicans* attenuates virulence. Infect Immun **65:**3529-3538.

14. **Sanglard D, Hube B, Monod M, Odds FC, Gow NA.** 1997. A triple deletion of the secreted aspartyl proteinase genes *SAP4,* *SAP5,* and **SAP6** of *Candida albicans* causes attenuated virulence. Infect Immun **65:**3539-3546.

15. **Schild L, Heyken A, de Groot PW, Hiller E, Mock M, de Koster C, Horn U, Rupp S, Hube B.** 2011. Proteolytic cleavage of covalently linked cell wall proteins by *Candida albicans* Sap9 and Sap10. Eukaryot Cell **10:**98-109.

16. **Lermann U, Morschhauser J.** 2008. Secreted aspartic proteases are not required for invasion of reconstituted human epithelia by *Candida albicans*. Microbiology **154:**3281-3295.

17. **Nobile CJ, Andes DR, Nett JE, Smith FJ, Yue F, Phan QT, Edwards JE, Filler SG, Mitchell AP.** 2006. Critical role of Bcr1-dependent adhesins in *C. albicans* biofilm formation *in vitro* and *in vivo*. PLoS Pathog **2:**e63.
