# Supplementary material for: Candida albicans-Induced Epithelial Damage Mediates Translocation through Intestinal Barriers
Source: mBio. 2018 Jun 5;9(3):e00915-18. doi: 10.1128/mBio.00915-18 (PMC5989070; doi:10.1128/mBio.00915-18)
Supplement: TABLE S2 [file mbo003183909st2.docx]

**Table S2:** *C. albicans* mutants analyzed for cellular damage, impact on epithelial integrity and fungal translocation.

| **Gene** | **Mutant strain description** | **Gene function** | **References** |
| --- | --- | --- | --- |
| ***CPH1/EFG1*** | Non-filamentous strains | Regulator of hyphal morphogenesis | Lo *et al.* 1997 (1)  Wartenberg *et al*. 2014 (2) |
| ***HGC1*** |  | Regulator of hyphal morphogenesis | Zheng *et al.* 2004 (3) |
| ***EED1*** | Mutants with characterized phenotypical defect | Regulator of hyphal elongation | Zakikhany *et al.* 2007 (4)  Martin *et al.* 2011 (5)  Polke *et al*. 2017 (6) |
| ***BRG1*** |  | Regulator of hyphal and biofilm formation | Lu *et al.* 2012 (7) |
| ***ALS3*** |  | Adhesin, invasin | Nobile *et al*. 2006 (8)  Liu & Filler 2011 (9) |
| ***BAS1*** |  | Putative transcription factor | Noble *et al*. 2010 (10)  Wangsanut *et al*. 2017 (11) |
| ***SNT1*** |  | Histone deacetylase | Noble *et al*. 2010 (10)  Nobile *et al.* 2014 (12) |
| **orf19.3335** | Damage-defective mutants | Plasma membrane protein | Skrzypek *et al*. 2017 (13) |
| ***TEA1*** |  | Transcription factor | Skrzypek *et al*. 2017 (13) |
| ***HMA1*** |  | Unknown | Skrzypek *et al*. 2017 (13) |
| ***AAF1*** |  | Regulatory protein | Skrzypek *et al*. 2017 (13)  Fu *et al*. 1998 (14) |
| ***PEP12*** |  | t-SNARE | Palanisamy *et al*. 2010 (15) |
| ***NPR2*** |  | Urea transporter | Skrzypek *et al*. 2017 (13) |
| **orf19.2797** |  | Unknown | Skrzypek *et al*. 2017 (13) |
| ***ECE1*** | *ECE1* related strains | *ECE1* deletion mutant | Moyes *et al*. 2016 (16) |
| ***KEX1*** |  | Intracellular peptidase | Moyes *et al*. 2016 (16) |
| ***SAP1-3*** | *SAP* related mutant strains | Secreted aspartic proteases | Kretschmar *et al.* 2002 (17)  Naglik *et al*. 2008 (18) |
| ***SAP4-6*** |  | Secreted aspartic proteases | Kretschmar *et al.* 2002 (17)  Naglik *et al*. 2008 (18) |
| ***SAP9-10*** |  | Surface-anchored aspartic proteases | Schild *et al*. 2011 (19) |
| ***SAP5*** |  | Secreted aspartic protease | Lermann *et al.* 2008 (20)  Villar *et al*. 2007 (21) |

1. **Lo HJ, Kohler JR, DiDomenico B, Loebenberg D, Cacciapuoti A, Fink GR.** 1997. Nonfilamentous *C. albicans* mutants are avirulent. Cell **90:**939-949.

2. **Wartenberg A, Linde J, Martin R, Schreiner M, Horn F, Jacobsen ID, Jenull S, Wolf T, Kuchler K, Guthke R, Kurzai O, Forche A, d'Enfert C, Brunke S, Hube B.** 2014. Microevolution of *Candida albicans* in macrophages restores filamentation in a nonfilamentous mutant. PLoS Genet **10:**e1004824.

3. **Zheng X, Wang Y, Wang Y.** 2004. Hgc1, a novel hypha-specific G1 cyclin-related protein regulates *Candida albicans* hyphal morphogenesis. EMBO J **23:**1845-1856.

4. **Zakikhany K, Naglik JR, Schmidt-Westhausen A, Holland G, Schaller M, Hube B.** 2007. In vivo transcript profiling of *Candida albicans* identifies a gene essential for interepithelial dissemination. Cell Microbiol **9:**2938-2954.

5. **Martin R, Moran GP, Jacobsen ID, Heyken A, Domey J, Sullivan DJ, Kurzai O, Hube B.** 2011. The *Candida albicans*-specific gene *EED1* encodes a key regulator of hyphal extension. PLoS One **6:**e18394.

6. **Polke M, Sprenger M, Scherlach K, Alban-Proano MC, Martin R, Hertweck C, Hube B, Jacobsen ID.** 2017. A functional link between hyphal maintenance and quorum sensing in *Candida albicans*. Mol Microbiol **103:**595-617.

7. **Lu Y, Su C, Liu H.** 2012. A GATA transcription factor recruits Hda1 in response to reduced Tor1 signaling to establish a hyphal chromatin state in *Candida albicans*. PLoS Pathog **8:**e1002663.

8. **Nobile CJ, Andes DR, Nett JE, Smith FJ, Yue F, Phan QT, Edwards JE, Filler SG, Mitchell AP.** 2006. Critical role of Bcr1-dependent adhesins in *C. albicans* biofilm formation *in vitro* and *in vivo*. PLoS Pathog **2:**e63.

9. **Liu Y, Filler SG.** 2011. *Candida albicans* Als3, a multifunctional adhesin and invasin. Eukaryot Cell **10:**168-173.

10. **Noble SM, French S, Kohn LA, Chen V, Johnson AD.** 2010. Systematic screens of a *Candida albicans* homozygous deletion library decouple morphogenetic switching and pathogenicity. Nat Genet **42:**590-598.

11. **Wangsanut T, Ghosh AK, Metzger PG, Fonzi WA, Rolfes RJ.** 2017. Grf10 and Bas1 Regulate Transcription of Adenylate and One-Carbon Biosynthesis Genes and Affect Virulence in the Human Fungal Pathogen *Candida albicans*. mSphere **2**.

12. **Nobile CJ, Fox EP, Hartooni N, Mitchell KF, Hnisz D, Andes DR, Kuchler K, Johnson AD.** 2014. A histone deacetylase complex mediates biofilm dispersal and drug resistance in *Candida albicans*. MBio **5:**e01201-01214.

13. **Skrzypek MS, Binkley J, Binkley G, Miyasato SR, Simison M, Sherlock G.** 2017. The Candida Genome Database (CGD): incorporation of Assembly 22, systematic identifiers and visualization of high throughput sequencing data. Nucleic Acids Res **45:**D592-D596.

14. **Fu Y, Filler SG, Spellberg BJ, Fonzi W, Ibrahim AS, Kanbe T, Ghannoum MA, Edwards JE, Jr.** 1998. Cloning and characterization of *CAD1*/*AAF1*, a gene from *Candida albicans* that induces adherence to endothelial cells after expression in *Saccharomyces cerevisiae*. Infect Immun **66:**2078-2084.

15. **Palanisamy SK, Ramirez MA, Lorenz M, Lee SA.** 2010. *Candida albicans PEP12* is required for biofilm integrity and *in vivo* virulence. Eukaryot Cell **9:**266-277.

16. **Moyes DL, Wilson D, Richardson JP, Mogavero S, Tang SX, Wernecke J, Hofs S, Gratacap RL, Robbins J, Runglall M, Murciano C, Blagojevic M, Thavaraj S, Forster TM, Hebecker B, Kasper L, Vizcay G, Iancu SI, Kichik N, Hader A, Kurzai O, Luo T, Kruger T, Kniemeyer O, Cota E, Bader O, Wheeler RT, Gutsmann T, Hube B, Naglik JR.** 2016. Candidalysin is a fungal peptide toxin critical for mucosal infection. Nature **532:**64-68.

17. **Kretschmar M, Felk A, Staib P, Schaller M, Hess D, Callapina M, Morschhauser J, Schafer W, Korting HC, Hof H, Hube B, Nichterlein T.** 2002. Individual acid aspartic proteinases (Saps) 1-6 of *Candida albicans* are not essential for invasion and colonization of the gastrointestinal tract in mice. Microb Pathog **32:**61-70.

18. **Naglik JR, Moyes D, Makwana J, Kanzaria P, Tsichlaki E, Weindl G, Tappuni AR, Rodgers CA, Woodman AJ, Challacombe SJ, Schaller M, Hube B.** 2008. Quantitative expression of the *Candida albicans* secreted aspartyl proteinase gene family in human oral and vaginal candidiasis. Microbiology **154:**3266-3280.

19. **Schild L, Heyken A, de Groot PW, Hiller E, Mock M, de Koster C, Horn U, Rupp S, Hube B.** 2011. Proteolytic cleavage of covalently linked cell wall proteins by *Candida albicans* Sap9 and Sap10. Eukaryot Cell **10:**98-109.

20. **Lermann U, Morschhauser J.** 2008. Secreted aspartic proteases are not required for invasion of reconstituted human epithelia by *Candida albicans*. Microbiology **154:**3281-3295.

21. **Villar CC, Kashleva H, Nobile CJ, Mitchell AP, Dongari-Bagtzoglou A.** 2007. Mucosal tissue invasion by *Candida albicans* is associated with E-cadherin degradation, mediated by transcription factor Rim101p and protease Sap5p. Infect Immun **75:**2126-2135.
